# Canonical and truncated transglutaminase-2 regulate mucin-1 expression and androgen independency in prostate cancer cell lines

**DOI:** 10.1038/s41419-023-05818-9

**Published:** 2023-05-09

**Authors:** Adeola Grace Atobatele, Elisa Tonoli, Jayakumar Vadakekolathu, Maria Pia Savoca, Melissa Barr, Yukti Kataria, Marta Rossanese, Izhar Burhan, Stephanie McArdle, Daniela Caccamo, Elisabetta A. M. Verderio

**Affiliations:** 1 School of Science and Technology, Centre for Health, Ageing and Understanding of Disease, Nottingham Trent University, Nottingham, NG11 8NS UK; 2 John van Geest Cancer Research Centre, Nottingham Trent University, Clifton Lane, Nottingham, NG11 8NS UK; 3 Department of Human and Paediatric Pathology, Polyclinic Hospital University, Via C. Valeria 1, 98125 Messina, Italy; 4 Department of Biomedical Sciences, Dental Sciences & Morpho-Functional Imaging, Polyclinic Hospital University, Via C. Valeria 1, 98125 Messina, Italy; 5 Biological Sciences Department (BiGeA), University of Bologna, Bologna, 40126 Italy; 6 Present Address: Department of Biological and Biomedical Sciences, Science Centre, School of Health, Science and Wellbeing, Staffordshire University, Leek Road, Stoke-on-Trent, ST4 2DF UK

**Keywords:** Cancer, Cancer genomics

## Abstract

Androgen independency is associated with poor prostate cancer (PCa) survival. Here we report that silencing of transglutaminase-2 (TG2) expression by CRISPR-Cas9 is associated with upregulation of androgen receptor (AR) transcription in PCa cell lines. Knockout of TG2 reversed the migratory potential and anchorage independency of PC3 and DU145 cells and revealed a reduced level of mucin-1 (MUC1) RNA transcript through unbiased multi-omics profiling, which was restored by selective add-back of the truncated TG2 isoform (TGM2_v2). Silencing of AR resulted into increased MUC1 in TG2KO PC3 cells showing that TG2 affects transcriptional regulation of MUC1 via repressing AR expression. Treatment of PC3 WT cell line with TG2 inhibitor ZDON led to a significant increase in AR expression and decrease in MUC1. ZDON also blocked the formation of MUC1-multimers labelled with TG amine-donor substrates in reducing conditions, revealing for the first time a role for TG2, which we show to be externalised via extracellular vesicles, in MUC1 stabilisation via calcium-dependent transamidation. A specific antibody towards TGM2_v2 revealed its restricted nuclear location compared to the canonical long form of TG2 (TGM2_v1), which is predominantly cytosolic, suggesting that this form contributes to the previously suggested TG2-mediated NF-κB activation and AR transcriptional repression. As TGM2_v2 transcription was increased in biopsies of early-stage prostate adenocarcinoma (PRAD) patients compared to subjects presenting inflammatory prostatitis, and total TG2 protein expression significantly increased in PRAD versus normal tissue, the role of TG2 and its truncated form as a prostate malignancy marker is suggested. In conclusion, this investigation has provided the first unbiased discovery of a novel pathway mediated by TG2 via MUC1, which is shown to contribute to androgen insensitivity and malignancy of PCa cells and be upregulated in PCa biopsies, with potential relevance to cancer immune evasion.

## Introduction

Malignant prostate tissue transformation is a multiple-step process evolving into metastatic cancer, with bone metastasis and involvement of soft tissue accounting for most treatment failure [1–3]. To establish bone or other metastasis, PCa cells undergo epithelial to mesenchymal transition (EMT) to detach in body-circulating tumour cells and then colonise new matrices. Cellular migration and adhesion promote invasion in the bone endosteum, and inclusion of integrins in tumour-derived exosomes has been suggested to participate in this process [4]. Once formed, PCa bone metastasis constitutes a highly dynamic system involving tumour cells, osteoclasts and osteoblasts leading to gradual bone damage and destruction [5]. Androgen receptor (AR) is a ligand-activated transcription factor controlling a variety of cellular events implicated in tumour progression and AR signalling is central to PCa [6]. Hormonal deprivation therapy improves the survival of PCa patients, however, androgen receptor mutations often accompany tumour progression up to the point when cancer progresses to an androgen-independent state known as castration-resistant prostate cancer (CRPC), affecting nearly 20–30% of PCa patients. Therefore, androgen responsiveness is key to successful PCa treatment [7] and low AR or AR negativity is typical of an aggressive type of PCa not responsive to androgen deprivation therapy, as such a major cause of PCa-associated death [8–11]. Bone metastases occurs in about 90% of men with CRPC. With little effect on long-term survival of current treatment plans, the need to gain insight into the molecular mechanism that influence the progression of the metastatic disease is essential to discover new therapeutic targets [8, 12–15].

Aberrant expression of transglutaminase-2 (TG2), a mediator of calcium-dependent post-translational modifications via transamidation of proteins, has been implicated in the progression of cancer. Either its increased expression in metastatic cells or dysregulation of the alternative splice forms of TG2 has been reported in different cancer types [16–25]. The mRNA of TG2 undergoes alternative splicing resulting in several transcripts, with the canonical form known as TGM2_v1, and the truncated variant TGM2_v2 regarded as predominant [26]. Although the majority of studies have linked an increase in TG2 with the survival, motility, invasion and drug/chemotherapy resistance of cancer, the role of the alternative splice forms has not been fully understood; however, dysregulation of the alternative splice forms in different cancer types has been reported [26].

Previous work has shown that TG2 causes transcriptional repression of AR via the binding of TG2-NF-κB complex to a cognate NF-κB binding site on the androgen receptor promoter [27]. Current thinking ascribes to TG2 the ability to associate with the NF-κB negative regulator IĸBα, leading to the degradation of IĸBα, thus resulting in the liberation of the active p65/p60 NF-κB complex in the cytosol [27]. The p65/RelA subunit of NF-κB is then translocated to the nucleus in complex with TG2 leading to a transcriptional repression of AR [27].

Since the truncated TG2 isoform escapes GTP inhibition and expresses a unique alternative C-terminal peptide, we hypothesise its participation in the steps leading to PCa progression and androgen independency of metastatic disease. Here we show that editing of TGM2 gene by CRISPR-Cas9 in two metastatic PCa cell lines (PC3 and DU145) reverses the metastatic PCa cell phenotype, and that selective knock-in of the truncated TG2 isoform leads to specific transcriptomics, uncovering a new link between TGM2_v2 and the oncoprotein Mucin-1 (MUC1) [28] which is mediated by AR downregulation. Consistent with its key molecular role, TGM2_v2 transcript has emerged as significantly overexpressed in PCa patients’ biopsies compared to prostatitis when confronted with the canonical long form of TG2.

## Results

### TG2 expression and activity correlate with androgen sensitivity in prostate cancer cell lines

**Fig. 1:**
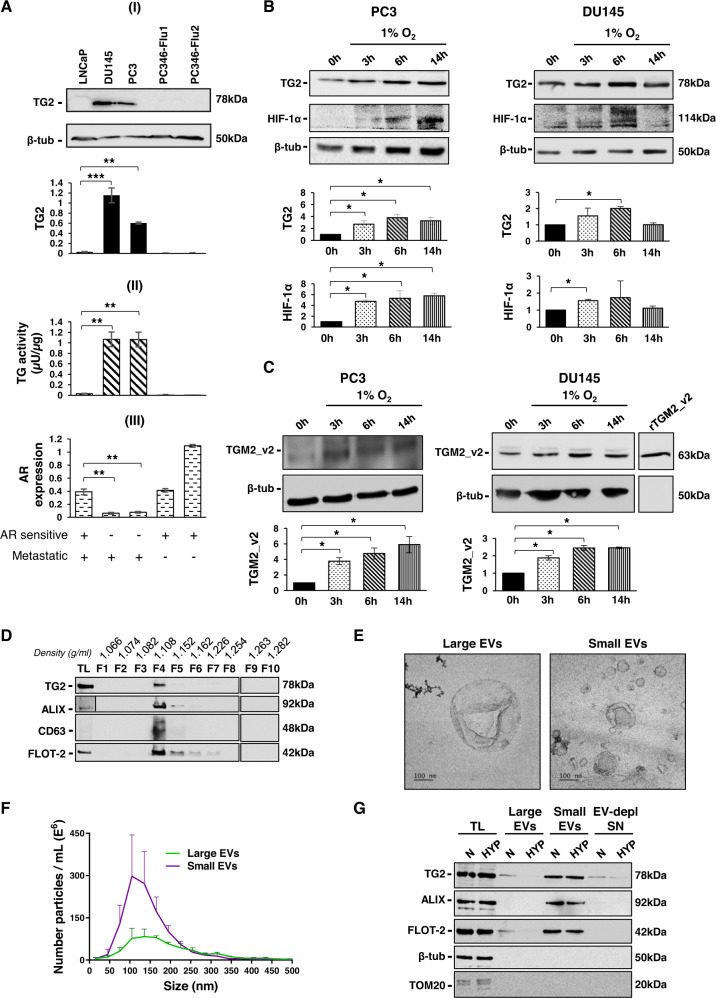
Characterisation of TG2 in AR-insensitive PCa cell lines and EVs under hypoxic conditions. **A** (I) Immunoblot showing expression of TG2 in androgen-sensitive and -insensitive PCa cell lines. Equal protein amounts of total cell lysates were immunoblotted and probed with mouse monoclonal anti-transglutaminase-2 (CUB7402) and rabbit polyclonal anti-β-tubulin as loading control. Densitometry analysis of immunoreactive bands was performed with intensity of band/area normalised to β-tubulin loading control. (II) TG activity was measured in total cell lysates by incorporation of biotinylated cadaverine into fibronectin (FN). Total TG activity is represented in µU/µg. (III) mRNA expression of androgen receptor (AR) using reverse-transcription polymerase chain reaction (RT-PCR) in PCa cell lines with tata-box protein (TBP) as a housekeeping control gene. All quantifications are the mean ± SD of three independent experiments, Student’s t-test: **p ≤ 0.01; ***p ≤ 0.001. **B**, **C** Immunoblot of total cell lysates from androgen insensitive metastatic PCa cells, PC3 and DU145 cultured in normoxic (37 °C, 5% CO_2_) and hypoxic (1% O_2_) culture conditions, probed using (**B**) mouse monoclonal anti-TG2 antibody (CUB7402) and mouse monoclonal anti-HIF-1α or (**C**) rabbit polyclonal anti-TGM2_v2 antibody and rabbit polyclonal anti-β-tubulin as a loading control. A representative blot is shown. Quantifications are the mean ± SD of three independent experiments; data are normalised to β-tubulin and to 0 h, Student’s t-test: *p ≤ 0.05. In (**C**), expression of a recombinant TGM2_v2 in *E. coli* served as a positive control. **D** Western blot analysis of EVs isolated from PC3 serum-free conditioned medium and processed by density gradient ultracentrifugation (Optiprep). The ten collected fractions were analysed for the detection of TG2, ALIX, CD63 and FLOT-2. PC3 WT total cell lysate (TL) was included to verify enrichment of EVs markers. **E** EVs characterization by Transmission electron microscopy (TEM). EVs were negative stained with uranyl acetate/methylcellulose. **F** EVs analysis by nanoparticle tracking analysis (NTA). The graph shows average particle concentration according to EVs size. **G** Western blot analysis of EVs and TCA-precipitated EV-depleted supernatants (EV-depl SN) isolated from PC3 cells under normoxic (N) and hypoxic conditions (HYP) for the detection of EVs markers ALIX, FLOT-2 and EVs negative marker TOM20. TL: PC3 WT total cell lysate.

TG2 has been associated with tumorigenesis with its expression reported to be lower in primary tumours compared to drug-resistant and metastatic tumours [29–31]. Androgen-insensitive PCa metastatic cell lines PC3 and DU145 displayed canonical TG2 protein (Fig. 1A-I) capable of calcium-dependent transamidation (Fig. 1A-II), whereas androgen-sensitive PCa cell lines either metastatic cell line LNCaP or non-metastatic PC346-Flu1 and PC346-Flu2 cell lines lacked TG2 protein expression or transamidation activity (Fig. 1A-I, II). A lack of AR transcript was confirmed in PC3 and DU145 while metastatic LNCaP and primary PC346-Flu1, PC346-Flu2 displayed AR transcript (Fig. 1A-III). Our data confirm a negative correlation between TG2 and AR and additionally show that androgen sensitive cell lines of both metastatic and non-metastatic phenotype have low levels of TG2. To mimic the solid tumour microenvironment, hypoxia was simulated by growing cells in low oxygen conditions (1% O_2_). Hypoxia-inducible factor-1 (HIF-1α) was gradually expressed and was also accompanied by increased TG2 in both the androgen insensitive PC3 and DU145 cell lines (Fig. 1B). Small changes in TG2 expression were observed in androgen insensitive LNCaP post chemical induced hypoxia (Supplementary Fig. 1). These new data in PCa cells are consistent with the notion that TG2 is a transcriptional target of HIF-1α and enhances the survival of hypoxic tumour cell [23, 32, 33]. As we previously reported aberrant expression of a truncated form of TG2 (TGM2_v2) displaying a new epitope in multiple cancer cell lines [26], we raised an antibody towards the unique epitope (in exon 10b) (Supplementary Fig. 2) to evaluate the presence of this TG2 isoform in PC3 and DU145. An immunoreactive band at 63 kDa, consistent with TGM2_v2 expression was displayed by both cell types and was considerably increased by hypoxia conditions (Fig. 1C).

The secretion of TG2 from cells to the ECM has been implicated in outside-in signalling in several cancer models [16, 22, 34], and recently TG2 has been proposed to be externalised via microvesicles/extracellular vesicles (EVs) in cancer [35–37] and other systems [38–40]. The presence of extracellular TG2 in EVs isolated from PC3 serum-free conditioned medium (CM), was demonstrated by density gradient, which showed TG2 in the EVs-rich fraction 4 where all the tested EVs markers were also concentrated (ALIX, CD63 and FLOT-2) (Fig. 1D). EVs were further characterised by TEM and NTA confirming the expected cup-shape morphology (Fig. 1E) and size range of large EVs (157.8 ± 21.9 nm) and small EVs (130.5 ± 8.2 nm) (Fig. 1F). TG2 was prominent in the small EVs but also present in the large EVs (Fig. 1G), however, the short isoform was negligible in both EVs fractions (Supplementary Fig. 3) Hypoxia did not increase the level of externalised TG2, however, serum-starvation, applied to avoid serum EVs contamination, would limit protein expression (Fig. 1G). A small level of TG2 was also found in soluble form in the EVs-depleted CM (Fig. 1G). Mass spectrometry of PC3-derived EVs confirmed the presence of TG2 protein in the total lysate and membrane fraction (Supplementary Tables 1 and 2), confirming for the first time that TG2 is externalised via EVs in the PC3 cells model.

### TG2KO impairs cell-matrix interaction in metastatic prostate cancer cell lines

**Fig. 2:**
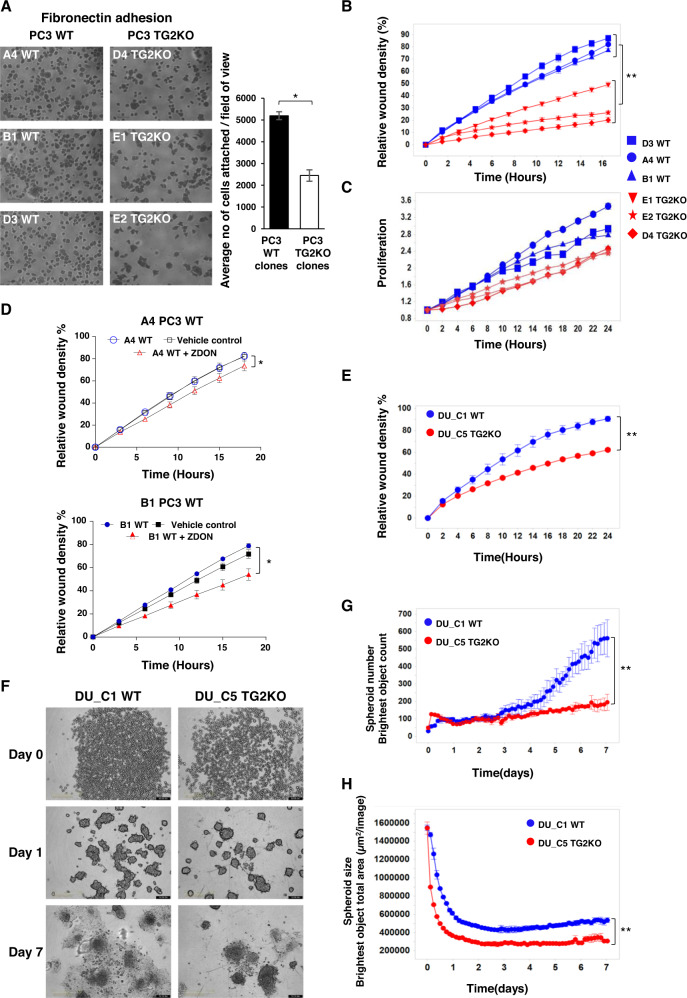
TG2KO decreases cell adhesion, migration and spheroid formation of metastatic PC3 and DU145 cells. **A** Cell attachment for 1 h on fibronectin of WT and TG2KO PC3 clones evaluated by manual counting of the number of attached cells from three fields of view (FOV) per clone. Data represent the mean of three independent experiments performed in quadruplicates. **B** Cell migration assay of three PC3 WT and three TG2KO clones grown to confluence (40,000 cells/well) in ImageLock 96-well plates and wound created using WoundMaker®. Time course of wound closure is expressed as relative wound density (%), which is a measure of cell density in the wound area relative to the density of cells outside of the wound area. Data are representative of three independent experiments performed in quadruplicates. **C** PC3 WT and TG2KO clones were grown on standard 96-well plates to assess proliferation. Time lapse course of proliferation is expressed as phase area confluence. Data normalised to the start of experiment, 0 h, *p* ≥ 0.18 (ns). **D** Cell migration assay of PC3 WT (A4 and B1 clones) treated with irreversible TG2 inhibitor ZDON and vehicle control (DMSO), to assess the effect on migration. The wound healing assay was performed as described in (**B**). **E** Cell migration assay of DU145 WT and TG2KO clones (DU_C1 WT and DU_C5 TG2KO) performed as described in (**B**). **F** Spheroid formation assay representative images of same DU145 WT and TG2KO clones. Cells were seeded in ultra-low attachment plate at 4000 cells/well, centrifuged and spheroid formation monitored using the IncuCyte® S3 live cell imaging from day 0 to day 7. **G** Reduced number of spheroids and **H** decreased size of spheroids formed by DU_C5 TG2KO compared to DU_C1 WT clone. Error bars represent ± SEM. Student’s t-test: *p ≤ 0.05; **p ≤ 0.01.

To explore the role of TG2 in the migration and adhesive ability of androgen insensitive metastatic cells, the TGM2 gene was edited to delete its expression using CRISPR-Cas9 and a guide RNA towards its first exon (Supplementary Fig. 4). Three stable PC3 TG2KO clones and three DU145 TG2KO clones were generated with null expression of TG2 and negligible calcium-dependent transamidation, and a matching number of control clones with wild-type levels of TG2 (Supplementary Fig. 5A, B). TG2KO was not compensated by other main transglutaminases when investigated in TG2KO clone E2 (Supplementary Fig. 6). Since bone metastasis is a more frequent outcome of androgen insensitive PCa, the role of TG2 was initially investigated in the PC3 TG2KO clones and wild-type clones (Supplementary Fig. 5A). Deletion of TG2 led to reduced fibronectin cell adhesion of serum-starved cells (Fig. 2A) and impaired wound migration (Fig. 2B) in the three TG2KO clones monitored in real time. There was no significant change in cell proliferation (Fig. 2C). Incubation of wild-type clones (A4 PC3 WT and B1 PC3 WT) with a specific inhibitor of TG2 transamidating activity (ZDON) led to a small but significant decrease in wound migration (Fig. 2D) suggesting that TG2 transamidation is implicated in the TG2 role. Knockout of TG2 in DU145 clones led to a similar change in wound migration (DU_C5 TG2KO and DU_C1 WT) (Fig. 2E). As shown in Fig. 2F, G, DU_C5 TG2KO clone formed significantly fewer anchorage-independent spheroids than DU_C1 WT clone (day 7). Moreover, DU_C5 TG2KO clone formed significantly smaller spheroids than DU_C1 WT clone (day 5) (Fig. 2H). The spheroids formed by DU_C1 WT clones started to spread into the surrounding environment at an earlier stage (day 7) relative to DU_C5 TG2KO (Fig. 2F). Therefore, silencing of TG2 impairs key tumorigenic processes including migration and anchorage-independent cell growth in two androgen-insensitive metastatic cell lines.

### TG2-isoform-dependent transcriptome in PC3 cells

**Fig. 3:**
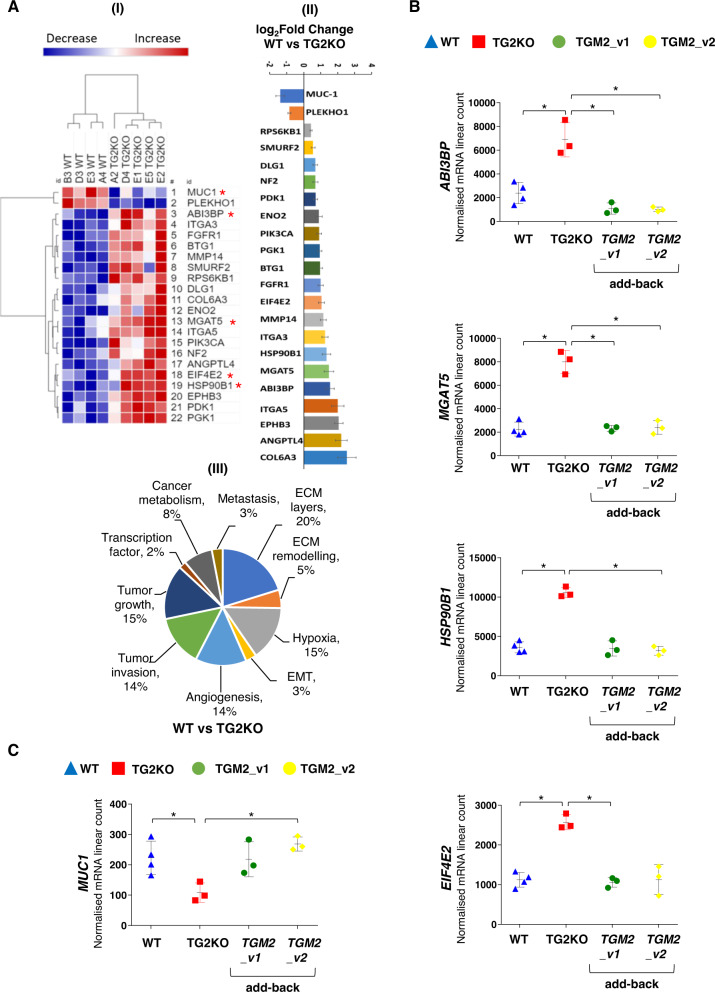
TG2-dependent expression of cancer-associated genes. **A** (I) Normalised mRNA log_2_ count of each gene modulated either by knockout of TGM2 or add-back of either isoform of TGM2 using NanoString Cancer progression panel, data plotted using Morpheus from Broad Institute https://software.broadinstitute.org/morpheus/. Hierarchical clustering and One minus Pearson Correlation in rows and columns. Intense blue to less intense blue signifies range of gene under expressed, while light red to intense red signifies range of gene over expressed as indicated in the key. Red asterisks indicate genes restored after add-back of TGM2_v1 and/or TGM2_v2. (II) log_2_ Fold change representation of genes upregulated and downregulated in PC3 TG2KO clones compared to WT PC3 clones. (III) Pie chart categorising the differentially expressed genes into cancer progression categories according to NanoString Cancer progression panel. Each cancer progression category shown with the percentage of gene hit against total number of cancer progression categories. **B**, **C** Five genes significantly restored up to WT levels upon add-back of TGM2_v2 and/or TGM2_v1. Scatter plots of normalised mRNA linear count for each differentially expressed gene (mean ± SD). Each symbol represents a clone. Blue triangles represent each WT clone, the red squares represent each TG2KO clone, green circle represents TGM2_v1 add-back clone, yellow circle represents TGM2_v2 add-back clone. Benjamini–Hochberg (BH) multiple correction test: *p ≤ 0.05.

The individual mRNA transcripts of 770 genes related to cancer progression were measured by digital transcriptomics, directly in the RNA isolated from the PC3 WT clones and individual PC3 TG2KO clones. TG2KO led to significant higher levels in the RNA transcript of 20 genes (Fig. 3A and Supplementary Table 3), a large proportion of which were related to extracellular matrix-cell interactions (Supplementary Table 3). Of the genes overexpressed upon TG2 silencing, only four transcripts (ABI3BP, MGAT5, HSP90B1 and EIF4E2) were compensated by add-back of either TGM2_v1 or TGM2_v2 in a significant manner (Fig. 3B). TG2KO led to significant lower levels in RNA transcript of two genes, consistently in all the clones analysed (Fig. 3A). These were MUC1, an oncoprotein associated with PCa progression forming protective mucous barriers on epithelial surfaces [28, 41, 42], and PLEKHO1 (Pleckstrin homology domain containing, family O member 1), known to be involved in EMT (Fig. 3A). Only MUC1 RNA transcript was compensated consistently in three individual TG2KO clones by adding back the truncated isoform TGM2_v2. Add-back of the TGM2_v1 isoform, the main long TG2 transcript (Fig. 3C), increased MUC1 transcript but not in a significant way. More genes were specifically induced by either TGM2_v1 or TGM2_v2 add-back or both (Supplementary Fig. 7A–C and Supplementary Table 4 and 5), but they were not significantly affected by TG2KO in a consistent way among all the analysed TG2KO clones. Functional categorisation (Supplementary Fig. 7D) did not reveal major differences between the transcriptome induced by the long TG2 isoform TGM2_v1 compared to the truncated isoform TGM2_v2, which shared approximately half of the induced transcripts. However, TGM2_v2 mediated the repression of over 20% of these cancer-related gene transcripts, among which is HSP90B1, and induced the expression of MUC1 (Supplementary Fig. 7B, C). HSP90B1 has been associated with androgen dependency in LNCaP and not in PC3 and DU145 [43] and MUC1 is an oncoprotein preferentially expressed in androgen insensitive PC3 and DU145 [28] suggesting that TG2-driven regulation of gene expression is aligned with its role in controlling AR.

### TG2 regulates MUC1 expression via repression of the AR and generates MUC1 multimers in cultured cells

It is known that AR suppresses MUC1 promoter via a consensus AR binding site [28], and as a consequence MUC1 is upregulated in androgen-independent PC3 and DU145 cells which have negligible AR. As TG2 promotes a decrease in AR expression [27] we hypothesise that TG2 participates in MUC1 regulation through transcriptional repression of AR and thus contributes to the aggressive PCa phenotype.

**Fig. 4:**
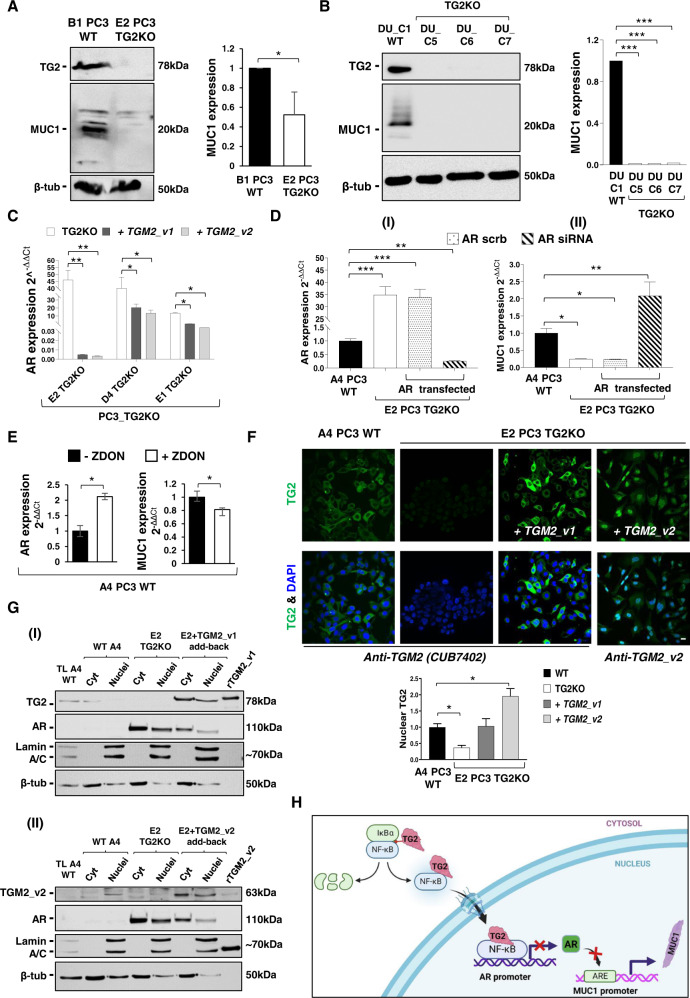
Modulation of MUC1 expression by TG2 via the androgen receptor in metastatic PCa cell lines. **A**, **B** Immunoblot showing decreased protein expression of MUC1 in TG2KO clones from metastatic PCa cell lines, PC3 and DU145. Densitometry analysis of MUC1 expression normalised versus β-tubulin loading control and WT cells, Student’s t-test: *p ≤ 0.05, ***p ≤ 0.001. **C** Relative quantifications of Androgen Receptor (AR) in three representative PC3 TG2KO clones (D4 PC3 TG2KO, E1 PC3 TGKO and E2 PC3 TG2KO), transfected back with either TGM2_v1 or TGM2_v2 in each clone. Values obtained from relative quantification of AR were normalised to the expression of AR in a WT clone (A4 PC3 WT). **D** Effect of AR knockdown on MUC1 expression. Relative expression of AR (I) and MUC1 (II) following siRNA knockdown of AR 48 h post transfection in E2 PC3 TG2KO clone compared to A4 PC3 WT. House-keeping gene, HPRT. **E** AR and MUC1 expression following 24 h pharmacological inhibition of TG2 using irreversible TG2 inhibitor ZDON (90 µM) in A4 PC3 WT clone. All qRT-PCR were analysed using Student’s t-test: *p ≤ 0.05, **p ≤ 0.01. **F** Nuclear localisation of TGM2_v2 protein in E2 PC3 TG2KO clone after being transiently transfected, as shown by immunofluorescence. E2 PC3 TG2KO cells transfected with pcDNA3.1(+)ValTGM2_v1 and pcDNA3.1/Hygro(-)TGM2_v2 for 48 h via electroporation. Cells were washed and fixed with ice-cold 90% methanol and incubated with primary antibody (either mouse monoclonal CUB7402, 1:200 or custom-made rabbit polyclonal anti-TGM2_v2, 1:200) for 16 h, followed by incubation with respective secondary antibodies conjugated with FITC, 1:200. Cells were then visualised using Leica SP5 confocal microscope. Quantification of nuclear staining using Image J Fiji software, Student’s t-test: *p ≤ 0.05. **G** Cytosolic and nuclear fractions of A4 PC3 WT clone, E2 PC3 TG2KO clone either transfected or not with TGM2_v1 (I) or TGM2_v2 (II) cDNAs were fractionated using the NE-PER nuclear and cytoplasmic extraction reagents kit (Thermo Scientific). Equal amounts (35 µg) of A4 WT total cell lysate (TL) and fractions were separated in a 8% SDS-polyacrylamide gel under reducing conditions and membranes probed with anti-TG2 CUB7402 (I) or anti-TGM2_v2 antibody (II) and AR. Loading controls were lamin A/C for the nuclear fractions and β-tubulin for the cytosolic fractions. TL: total cell lysate. **H** Schematic depiction of the mechanism of MUC1 regulated expression via TG2 and AR expression in PCa cells. In PCa cells TG2 leads to IĸBα degradation with release of its subunits (p65/p50) [27]. The TG2/p65/50 complex translocates into the nucleus [28] and inhibits AR expression: without AR the mucin-1 promoter is not blocked and this leads to MUC1 transcription. This schematic was created with BioRender.

MUC1 was revealed in multiple forms (20–25 kDa) by western blotting in PC3 WT lysates (B1 PC3 WT) and at a significantly lower level in PC3 TG2KO cells (E2 PC3 TG2KO shown) (Fig. 4A). The same trend was observed in DU145 TG2 WT cells (DU_C1 WT) which displayed higher MUC1 compared to the TG2KO clones (DU_C5, DU_C6 and DU_C7 TG2KO) (Fig. 4B). MUC1 multiple forms, resistant to reduction, could either be due to extensive O-linked glycosylation or to post-translational modification mediated by TG2 cross-linking.

In parallel, TG2KO resulted into an increased level of AR over WT control and reconstitution of either TGM2_v1 or TGM2_v2 resulted into a lowering of AR several folds (Fig. 4C) with no significant differences between the isoforms in PC3 clones. Silencing of AR by siRNA in the E2 PC3 TG2KO cells (Fig. 4D-I) restored low levels of AR expression compared to scrambled siRNA and resulted into several fold increase in MUC1 (Fig. 4D-II) suggesting that TG2 has a role in the transcriptional regulation of MUC1 via repressing AR expression. Treatment of PC3 WT cells (A4 PC3 WT) with TG2 inhibitor ZDON led to a significant increase in AR expression (Fig. 4E) and a decrease in MUC1 (Fig. 4E and Supplementary Fig. 8). Notably, the effect of ZDON was not as marked on both AR and MUC1 transcript decrease, as seen by gene deletion (Fig. 4A, C).

As MUC1 forms the mucosal surfaces of many tissues representing a protective layer against external insults and it is aberrantly expressed in cancer, we monitored whether TG2-mediated transamidation could alter MUC1 post-translationally as well as concur to its production. To do so two cell lines (PC3 and DU145 WT) were cultured in the presence of a primary amine TG substrate (FITC-cadaverine) to identify glutaminyl substrates in live cells. High Molecular Weight (HMW) multimers of MUC1 were consistently seen in reducing conditions, which were inhibited by a specific TG2 inhibitor (ZDON) (Supplementary Fig. 9A), suggesting that TG2 stabilises MUC1. In support of this, the dotted in situ TG activity monitored by FITC-cadaverine incorporation in live cells partly overlapped MUC1 immunofluorescence at the cell periphery and in the pericellular matrix of PC3 and DU145 clones, as shown by the yellow/orange merged spots (degree of colocalization 31–45%) (Supplementary Fig. 9B).

### TGM2_v2 is located in the nucleus

TG2 is an activator of nuclear factor (NF)-κB and it has been suggested that this occurs independently from the canonical IKK phosphorylation and Iκα degradation pathway, and leads to AR repression [23, 27]. This would imply translocation of TG2 to the nucleus together with the p65/RelA subunit of NF-κB, as previously shown [27]. Therefore, the localisation of TG2 was investigated by immunofluorescent staining in a control clone (A4 PC3 WT) and a TG2KO clone (E2 PC3 TG2KO) after add-back of TG2 with either the long (TGM2_v1) or the short isoform (TGM2_v2) by cell transfection. The immunostaining of TG2 with a specific antibody for either isoform (CUB7402) showed a diffuse staining of fixed and permeabilised cells, mainly cytosolic but with a weak nuclear signal (Fig. 4F). The cytosolic staining was sharper after TGM2_v1 add-back and clearly confined to this compartment. TGM2_v2 add-back resulted into a strong nuclear immunoreactivity when a polyclonal antibody raised against the TGM2_v2 unique epitope was applied (Fig. 4F).

These data were validated by western blotting. The wild-type PC3 cells (A4 PC3 WT) displayed immunoreactivity to TGM2_v2 (63 kDa band) predominantly in the nuclear fraction and not in the cytosolic fraction when equal protein amount was examined (Fig. 4G-II) and immunoreactivity to TGM2_v1 (78 kDa band) mainly in the cytosol (Fig. 4G-I). After TGM2_v2 or TGM2_v1 cDNAs add-back into the TG2KO cells (E2 PC3 TG2KO), TGM2_v2 was confirmed to be present in the nucleus although it was also expressed in the cytosol after cell transfection (Fig. 4G-II) and the overexpressed TGM2_v1 was located in the cytosol but also, at a lower extent, in the nucleus after forced expression (Fig. 4G-I). The loading controls revealed equal loading of the nuclear (lamin A/C control) and cytosolic fractions (β-tubulin control). Consistent with the qRT-PCR data (Fig. 4C), AR was absent in the WT PC3 cells, induced by TG2 knockout, and its nuclear expression inhibited again by TGM2_v2 (Fig. 4G-II) and also TGM2_v1 add-back (Fig. 4G-I). This shows that both transfected TG2 forms can modulate AR expression, however, the endogenous truncated TG2 form, by specific accumulation in the nucleus most likely leads to specific lowering of AR expression, thus conferring growth advantage to the cancer cells in this way.

Collectively these data suggest that TG2 concurs to PCa progression which induces androgen independency via repression of AR (Fig. 4G) leading to dysregulation of MUC1 expression, a feature of more aggressive PCa pathology.

### Positive correlation of TG2 and MUC1 expression in prostate cancer patients and expression of the TG2 variants

**Fig. 5:**
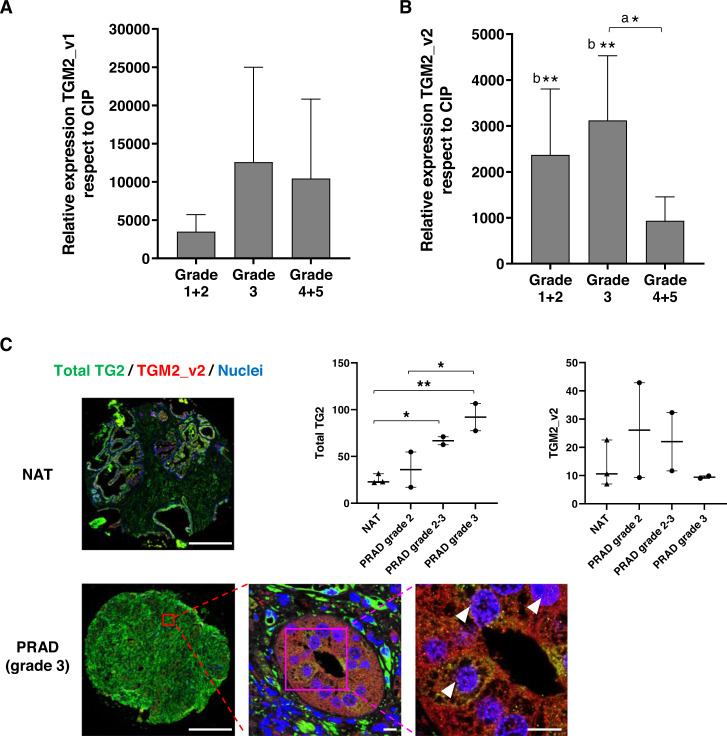
TG2 expression in PRAD biopsies compared to controls. Quantitative PCR of TGM2_v1 transcript (**A**) and TGM2_v2 transcript (**B**) in PRAD biopsies (*n* = 27) compared to CIP (*n* = 30). Data are mean + SEM and expressed as fold increase compared to CIP. Significant differences between PRAD grades (a) and PRAD in comparison with CIP (b) were assessed by one-way ANOVA. **C** Immunostaining of total TG2 protein (mouse anti-TG2 CUB7402, green), TGM2_v2 (rabbit anti-TGM2_v2, red) and nuclear staining (blue) in PRAD (*n* = 6) and normal adjacent tissue (NAT, *n* = 3) TMA (TissueArrays.com), using GeoMx platform (Nanostring Technologies). Scale bar: 0.5 mm. Box and whiskers plot shows all data points of total TG2 or TGM2_v2 intensity measured with ImageJ Fiji software and bars from min to max values. Significance was assessed by one-way ANOVA: *p ≤ 0.05, **p ≤ 0.01. The bottom panels show the detail of a grade 3 PRAD TMA acquired with a SP5 confocal microscope (63X objective; scale bars: 10 µm). White arrowheads indicate areas of TGM2_v2 nuclear localisation (pink spots).

Meta-analysis of TG2-MUC1 co-expression in seven publicly available PCa data sets showed a correlation between TG2 and MUC1 expression in five clinical studies for a total of 868 patients [44–47] validating the expression reported in PCa cell lines, indicating that the TG2-MUC1 axis could be a PCa hallmark (Supplementary Fig. 10). Next, we assessed the expression of both the canonical long form of TG2 (TGM2_v1) and the alternative truncated transcript TGM2_v2, in 57/101 patients’ biopsies after the exclusion of PCA3− biopsies from the prostate tumour group and PCA3+ biopsies from the prostate inflammation group. Expression of TGM2_v1 in prostate adenocarcinoma (PRAD) biopsies relative to Chronic Idiopathic Prostatitis (CIP), revealed a large trend increase associated with cancer progression, although not at a significant level due to sample variability (Fig. 5A). TGM2_v2 was several folds higher both in low-grade (Grade 1–2) and Grade 3 differentiated tumours compared with that in CIP (Fig. 5B), suggesting that TG2 may be a marker of malignancy. Moreover, meta-analysis of TGM2_v1 and TGM2_v2 transcripts from archived data (49 PCa and 12 benign tissues) [45] revealed a significant increased expression of 85% and 56%, respectively, in PCa tissue. Total TG2 protein expression was evaluated in PRAD tumour microarrays (TMA) and compared with normal adjacent tissue (NAT) (Fig. 5C). TG2 was prevalent in the tumour stromal tissue and significantly increased at grade 2–3 and 3 compared to NAT. Detection of the truncated TG2 isoform with the antibody raised against the specific epitope revealed its intracellular and intranuclear location consistent with a role in modulating gene expression. The truncated TG2 protein expression value was variable by antibody staining and did not increase significantly in PRAD versus NAT as opposed to its transcript by qRT-PCR (Fig. 5B). We conclude that TG2 is a PCa malignancy marker and that the TG2 isoforms may have different locations in the stroma and in the nucleus reflecting multiple pathogenetic roles.

## Discussion

This study uncovers upregulation of TG2 isoforms in metastatic androgen insensitive PCa cell lines in hypoxic conditions and in PRAD biopsies, which as such is emerging as a potential new marker of PCa. By dissecting cancer gene expression driven by the truncated TG2 isoform and the canonical one, an unbiased new link between TG2 and MUC1 has been established and regulation of MUC1 via transcriptional repression of AR by TG2 revealed for the first time. This finding explains, expands and gives mechanistic value to previous observations, with the same PCa cell types, that NF-κB is capable of binding the androgen receptor (AR) promoter leading to gene silencing. Although both TG2 isoforms are involved, we show in particular that the truncated form of TG2 which resides in the nucleus is likely a nuclear driver in AR suppression resulting into MUC1 upregulation.

The link between androgen independency and PCa aggressivity is well established [5–7]. In line with previous findings [27], this study has confirmed that an inverse correlation exists between AR expression and TG2 expression in metastatic PCa cell lines. Metastatic androgen insensitive PCa cell lines PC3 and DU145 displayed high TG2 upregulation, induced in conditions of HIF-1α expression, contrary to androgen sensitive metastatic cell line LNCaP and primary PCa lines. Moreover, hypoxia induced the expression of the truncated TG2 isoform (TGM2_v2) that unlike the canonical long form (TGM2_v1) lacks the C-terminal regulatory peptide which controls TG2 transamidation via GTP binding [23, 48, 49]. Several studies have proposed that the upregulation of TG2 in cancer is linked with unconventional activation of NF-κB [23, 50, 51], via degradation of IκBα in an IKK-independent way [23]. In particular, in PCa, NF-κB is capable of binding the AR promoter leading to AR silencing [27]. To gain insights in gene regulation dependent on TG2 isoforms, the long canonical TG2 form and the truncated dysregulated TG2 form were added back to a number of androgen insensitive PC3 clonal cell lines with stable TG2KO. Here we have shown that knockout of TG2 in androgen insensitive PCa cells leads to restoration of AR expression, which can be repressed again by the add-back of TG2 isoforms, either the long or the truncated form of TG2 in a significant way. In the same way, when cancer-related transcriptome was investigated using digital transcriptomics, MUC1 expression was revealed to be dependent on TG2 expression, since TG2KO lowered MUC1 expression and TG2 re-expression restored wild-type levels of MUC1; in particular the truncated TG2 isoform significantly restored MUC1 when transfected back in the TG2KO clones.

Other transcripts were also upregulated by TG2KO. Two encoding extracellular proteins ABI3BP (ABI Family Member 3 Binding Protein), involved with collagen ECM and cell adhesion, and MGAT5 (Alpha-1,6-Mannosylglycoprotein 6-Beta-N-Acetylglucosaminyltransferase) a glycosyltransferase involved in the regulation of the glycoprotein oligosaccharides biosynthesis affecting cell migrations of cells. Others include EIF4E2 (Eukaryotic Translation Initiation Factor 4E Family Member 2) and HSP90B1 of cytosolic location. However, MUC1 was the only transcript significantly upregulated by the aberrant TG2 isoform.

This is the first time that a link between TG2 and MUC1 was established. MUC1 is an oncoprotein overexpressed in human PCa the regulation of which is still being understood [28, 41, 52]. As a type I glycoprotein, MUC1 is a transmembrane member of the mucin family, a heterodimer consisting of a large N-terminal fragment (MUC1-N) and a C-terminal subunit (MUC1-C) and it is widely expressed on the apical surface of most epithelial tissues. In androgen-insensitive cell lines, PC3 and DU145 cells, MUC1 is highly expressed [28, 52]. On the other hand, androgen-sensitive cell lines including LNCaP, CWR22Rv1, MDA-PCa-2b do not express or have little MUC1, but express AR, suggestive of an inverse relationship between the two [28, 52]. Work by Kufe’s group has shown that transfection of AR reconstitutes low levels of MUC1 in PC3 and DU145 cells, due to AR occupying a consensus on the AR element of MUC1 promoter [52]. Here we have shown that TG2KO restores AR expression in PC3 cells and at the same time lowers MUC1 transcript. Therefore, TG2 participates in MUC1 regulation via transcriptional repression of AR. In particular, it is the aberrant form of TG2, the truncated one to accumulate in the nucleus and be involved in this process although full-length TG2 is likely to serve the same when forced in the nucleus. The reason for this would be that the short form of TG2 lacks the putative nuclear export signal motif at the C-terminus of TG2 at position 657 of the long form [53], resulting into prevailing import of the short TG2 via the nuclear localisation signal, which lies in the N-terminal beta-barrel-1 domain, hence its nuclear accumulation. A previous report showed nuclear accumulation of a truncated short form of TG2 (TG2-S) which we had recognised as TGM2_v2 [26], in a normal human hepatic cell line following ethanol and free fatty acid stress [54]. Although we have been able to reach similar conclusions by tracking TGM2_v2 protein expression by means of a recombinant protein obtained in house and an anti-v2 polyclonal antibody, the TGM2_v2 unique epitope is short, hampering its detection, which is also difficult when well-characterised antibodies such as CUB7402 targeting upstream epitopes are employed.

The TG2 role appears to depend on TG2 transamidation partially, since a well-characterised TG2 inhibitor (ZDON) recapitulates AR expression and MUC1 downregulation to some extent, although at a lower significance level than seen via TG2 gene silencing/add-back.

In a previous study in PC3 cells nuclear extracts, a complex between RelA (p65)/NF-κB heterodimer and TG2 was reported to colocalise in the nucleus implying a nuclear translocation on the TG2- NF-κB, leading to transcriptional repression of AR [27]. Here, we have observed a nuclear localisation of the aberrant form of TG2 (TGM2_v2) the expression of which regulates MUC1 in PC3 TG2KO cell lines. This important new finding suggests that the truncated form of TG2 which we have found expressed in PC3 and DU145 upon hypoxic stimulus may be involved in the TG2-mediated activation of NF-κB leading to AR repression and MUC1 overexpression, a pathway linked with PCa cells aggressivity.

Mucin-1 is aberrantly expressed in a number of human cancer types, including breast, ovarian, bladder, pancreatic, lung and prostate with loss of polarity in cancer cells, and differentially glycosylated [42, 52], with reports of poor survival, and poor response to therapy. MUC1 extracellular domain was reported to contribute to a decrease in the immune response in breast cancer cells [55]. The cytoplasmic C-terminal subunit has been attributed to its oncoprotein properties via signal transduction roles [42, 56, 57]. We show clinical significance in this by the positive correlation found between TG2 and MUC1 expression in PCa using archived data for five independent clinical studies. A link between the aberrant TGM2 transcript and PRAD was also established for the first time in this study when PRAD and CIP cohorts were compared. Furthermore, in PRAD TMA, TG2 protein increased overall compared to normal tissue and had an abundant stromal location, while the alternative TG2 epitope was tracked inside cells and in the nucleus.

Phenotypically, TG2 silencing in two androgen insensitive PCa cell lines led to clonal cell lines with impaired cellular adhesion and cell migration and anchorage-independent spheroid formation. Several lines of evidence have previously shown that TG2 plays a role promoting migratory ability of cancer cells including breast cancer, pancreatic cancer, epidermal and neuroblastoma cells [18, 58, 59]. In lung cancer, recent evidence suggests that TG2 promotes migration and invasion by positively regulating the activation of Rac [19]. All PC3 TG2KO clones displayed reduced cell migration post deletion of TG2 and the DU145 TG2KO clones displayed defects in spheroid formation, suggestive of a role of TG2 supporting migration and anchorage independency. In addition, inhibition of transamidating activity of TG2 pharmacologically showed reduced migration of metastatic PC3 cells suggesting that TG2 promotes cell migration through its cross-linking activity. We know that TG2 is present in the cytosol and in the nucleus [60, 61], but it is also released from cells via extracellular vesicles [35, 37–39] and here we have shown for the first time that the canonical form of TG2 is prominent in small extracellular vesicles of PC3 cells. Interestingly, we have found that EVs not only contain TG2 but also mucin isoforms in the membrane and intraluminal fractions that may be communicated from cell to cell. Thus, the externalisation of TG2, a leaderless protein, via small and large EVs is consistent with our recent observations that EVs are carriers of TG2 and concur to the accumulation of TG2 in the extracellular environment. As TG2 has a role in anchorage independency and cell migration in two metastatic PCa cell lines (PC3 and DU145), it is linked with the invasive phenotype and its secretion via EVs could be part of the mechanism.

The link between TG2 and MUC1 is novel. Very recently while this report was prepared, Recktenwald reported that epidermal transglutaminase-3 is able to cross-link and hence stabilise the gel forming mucus Mucin 2 with relevance in colon protection from inflammatory processes such as colitis [62]. We have revealed for the first time that MUC1 is also a cross-linking substrate for TG2. Therefore, TG2 participates in the mechanism of MUC1 overexpression in the nucleus, but MUC1 is also stabilised by TG2 at the surface of epithelial cells. It is tempting to speculate that TG2 could help stabilise a MUC1 barrier, potentially making PCa cells immune resistant. A coating of human pancreatic ductal carcinoma (PDA) by other filamentous proteins such as a keratin 19-CXCL2 complex stabilised by TG2 was recently proposed to evade the immune attack [63]. The expression of transglutaminase-4 (TG4), in PCa compared with benign counterparts has been debated with different studies leading to contrasting conclusions [64, 65]. Recently, TGM4 was identified as a potential prostate-specific antigen in castration-resistant luminal epithelial cells [66]. As TG4 is not strongly expressed in PC3 and DU145 [67, 68], neither TG2 appears to be compensated by TG4 following TG2KO, it is unlikely that TG4 would take part in this TG2-driven disease process.

In conclusion, this study has identified a novel pathway mediated by TG2 which may contribute to androgen insensitive PCa cells aggressivity and resistance via MUC1 upregulation and aberrant expression of truncated TG2 isoform. Understanding how MUC1 can be stabilised by TG2 and the relevance in cancer immune evasion will clarify the full significance of this new finding.

## Materials and methods

### Cell culture conditions

Metastatic PCa cell lines PC3, DU145, LNCaP were purchased from the ATCC Centre; non-metastatic PC346 Flu-1 [69] and PC346 Flu-2 [69] were kindly provided by Prof Jenster group (University Medical Center Rotterdam, the Netherlands). Growth conditions, supplements, antibodies and reagents are listed in supplementary methods.

### Extracellular vesicles (EVs) isolation and characterisation

EVs were isolated and characterised as previously described [39]. Briefly, cells were incubated in serum-free medium for 24 h. Conditioned medium was supplemented with protease inhibitors and serially centrifuged at 300 × *g* for 10 min (three times), 1200 × *g* for 20 min, 10,000 × *g* for 30 min to pellet large EVs and finally 110,000 × *g* for 70 min to pellet small EVs. EVs pellets were either lysed in RIPA buffer for western blotting analysis or resuspended in particle-free PBS for Nanoparticle Tracking Analysis (ZetaView PMX 120, Particle Metrix, Inning am Ammersee, Germany) and Transmission Electron Microscopy (JEM-2100Plus, JEOL, Herts, UK). The proteome of EVs lysates and EVs membrane fractions were subjected to reduction, alkylation and trypsin digestion, then analysed by RP-HPLC-ESI-MS/MS using a TripleTOF 6600 mass spectrometer (SCIEX, Ontario, Canada) in data-dependent acquisition mode [38]. Density gradient fractionation was performed as previously described [39].

### Generation of stable TG2 knockout cell lines by CRISPR-Cas9

HCP218061-CG01-1-B plasmid containing both sgRNA and Cas9 enzyme targeting *exon*-1 of human TGM2 gene (Genecopoeia, Rockville, MD, USA) was transfected in WT PC3 cells via electroporation (Amaxa Kit V, Lonza, Manchester, UK). Transfected cells were subjected to G418 (700 µg/ml) selection, and stable clones were picked for clonal expansion and screening. The TGM2 locus targeted by CRISPR-Cas9 was sequenced (Sanger sequencing) and validated for TG2KO. gDNA was isolated using DNeasy Blood & Tissue kit (Qiagen, Manchester, UK). Loss of TG2 protein was confirmed via western blotting (as described in the supplementary methods).

### Gene expression profiling using the NanoString nCounter™ FLEX platform

For Digital detection of RNA transcripts (nCounter, Nanostring, Seattle, WA, USA) the human PanCancer progression panel containing 770 genes was employed. Total RNA (150 ng), quality checked by Nanodrop and Qubit high sensitivity RNA, was used for Nanostring hybridisation according to the manufacturer’s instruction, at 65 °C for 20 h as previously described [70]. The transcriptomic data obtained from digital acquisition step (nCounter) were analysed and normalised using the nSolver™ software 4.0 (Advanced analysis module, version 2.0). Benjamini–Hochberg (BH) multiple correction test to the *p*-value was utilized to ensure exclusion of false discovery rate (FDR) where *p*-value of ≤0.05 was considered as significant. The differentially expressed genes were further visualised as a heatmap using Morpheus software (https://software.broadinstitute.org/morpheus/) and normalised log_2_ count of differentially expressed genes plotted.

### Analysis of TG2 transcripts in patients’ biopsies

TGM2_v1 and TGM2_v2 transcripts were quantified by Real-time qRT-PCR in prostate biopsies of patients affected by prostate adenocarcinoma (PRAD) or Chronic Idiopathic Prostatitis (CIP), as described in the supplementary methods.

### Statistical analysis

Student’s t-test and one-way ANOVA were used in experiments (two-tailed distribution, equal variance unless stated otherwise). A *p*-value equal to or less than 0.05 was considered as significant (*p ≤ 0.05, **p ≤ 0.01, ***p ≤ 0.001). Analysis of off-site target deletions via computational analysis, all of which were located in intronic regions of genomic locus, revealed a score of 0.1–1.3 as shown in Supplementary Table 8 [71].

## Supplementary information

Supplementary Information

Supplementary Table 1

Supplementary Table 2

Reproducibility checklist

Original Western blots

## Data Availability

All data generated or analysed during this study are included in this published article and its supplementary information files.
