# Supplementary material for: Canonical and truncated transglutaminase-2 regulate mucin-1 expression and androgen independency in prostate cancer cell lines
Source: Cell Death Dis. 2023 May 9;14(5):317. doi: 10.1038/s41419-023-05818-9 (PMC10170068; doi:10.1038/s41419-023-05818-9)
Supplement: Supplementary file 1 — Supplementary Information [file 41419_2023_5818_MOESM1_ESM.pdf]

Supplementary Information for

Canonical and truncated transglutaminase-2 regulate mucin-1 expression and androgen independency in prostate cancer cell lines.

Adeola Grace Atobatele, Elisa Tonoli, Jayakumar Vadakekolathu, Maria Pia Savoca, Melissa Barr, Yukti Kataria, Marta Rossanese, Izhar Burhan, Stephanie McArdle, Daniela Caccamo and Elisabetta AM Verderio.

Table of content:

- Supplementary Figures 1 to 10
- Supplementary Tables 3 to 9 (see separate files for supplementary Table 1 and 2)
- Supplementary Methods
- Supplementary References

Supplementary Figures

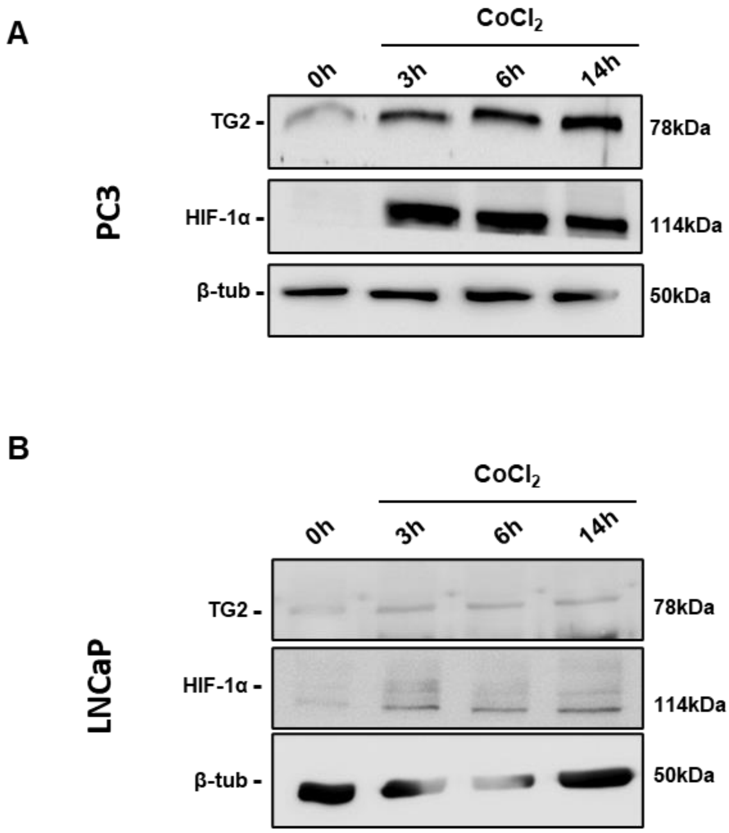

Supplementary Figure 1. TG2 expression in chemical hypoxic conditions in PC3 and LNCaP cells. (**A, B**) Immunoblot of total cell lysates from androgen insensitive (PC3) and androgen sensitive (LNCaP) metastatic cells in normoxic (37°C, 5% CO_2) and chemical hypoxic (100-150 µM $CoCl_2$) culture conditions, probed using mouse monoclonal anti-TG2 antibody (CUB7402), mouse monoclonal anti-HIF-1α and rabbit polyclonal anti-β-tubulin, as a loading control. Immuno-reactive bands were visualised via chemiluminescence detection system as described in Figure 1 legend.

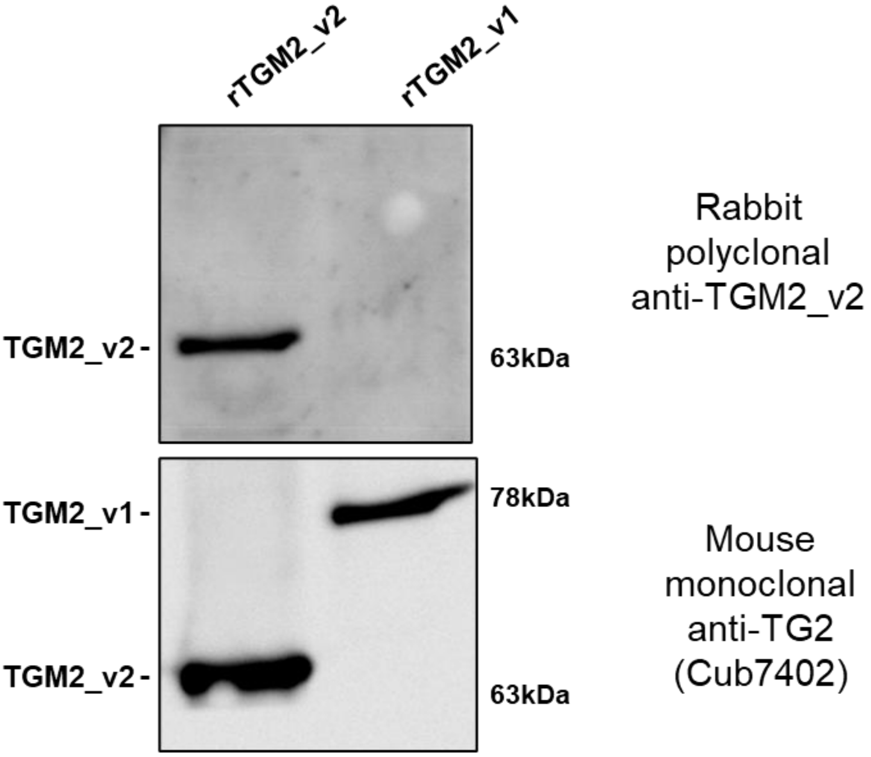

Supplementary Figure 2. Immunoreactivity of anti-TGM2_v2 and CUB7402 antibodies towards TG2 isoforms. Recombinant human TG2 isoforms expressed and purified as described in (1), were immunoprobed using either custom-made rabbit polyclonal anti-TGM2_v2 or mouse monoclonal anti-TG2 (CUB7402) antibody. Anti-TGM2_v2 recognises the truncated TG2 isoform only, whereas CUB7402 is able to recognise both isoforms.

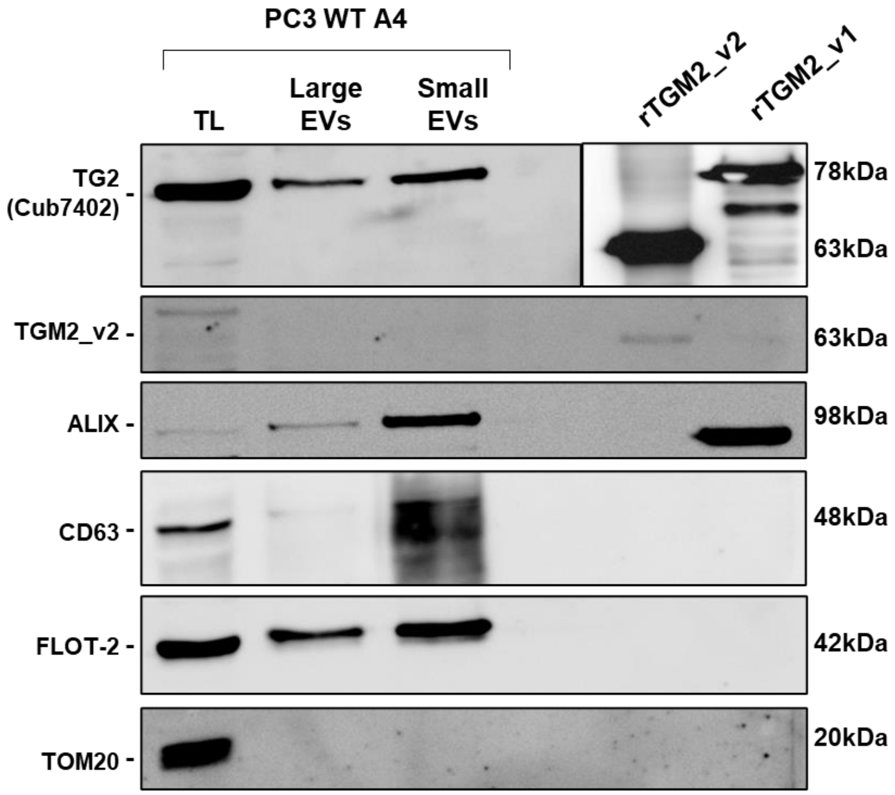

Supplementary Figure 3. Expression of TG2 isoforms in PC3-derived EVs. EVs were isolated from PC3 WT A4 cells (3 T75 flasks) and all material (15 µg for large EVs, 28 µg for small EVs and total cell lysate, TL) was loaded in a 10% polyacrylamide gel under reducing conditions and membranes probed with anti-TG2 CUB7402, anti-TGM2_v2 antibody, EVs markers (ALIX, CD63, FLOT-2) and negative EVs marker TOM20.

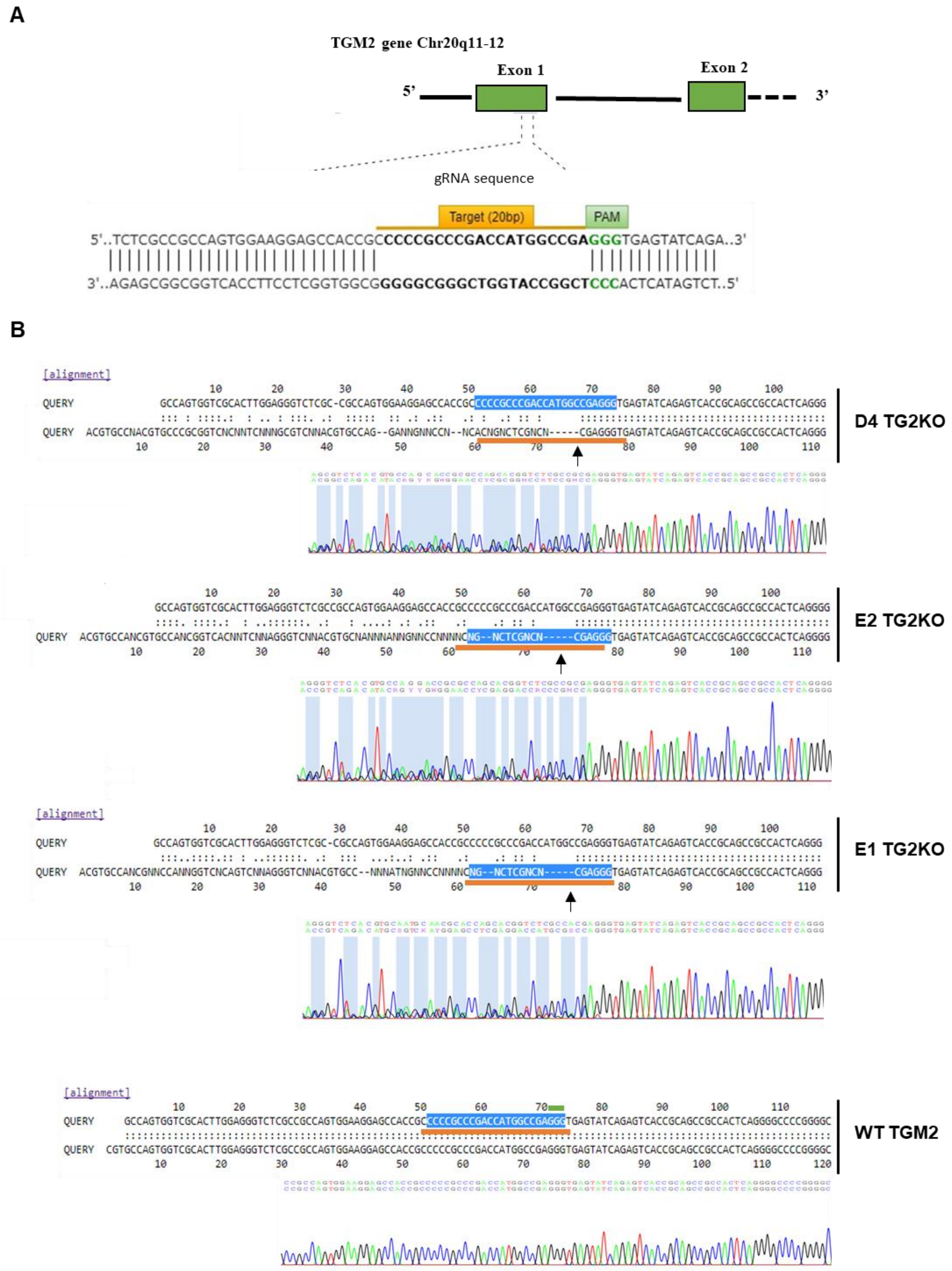

Supplementary Figure 4. CRISPR-Cas9 gene editing of TGM2 and validation. A) The single guide RNA (gRNA) target sequence (exon 1 of human TGM2) is highlighted followed by the protospacer adjacent motif (PAM) enabling cleavage by the CRISPR-Cas9. **B)** Sanger sequencing of PCR amplified TGM2 fragments encompassing the gene editing event. Sequence alignments of wild-type TGM2 (top sequence in each pair match) with the mutant

TGM2 gene sequences (bottom) isolated from the PC3 TG2KO clones (D4, E2 and E1) or wild type PC3. DNA sequencing was obtained by reverse primer 5’-CCTGGCGGACCACTGGCACGT -3’. The electropherograms (Sanger sequencing) are also shown. The black arrows point at the start of the shift in reading frame in the TG2KO clones (confirmed by western blotting, Supplementary Fig 4).

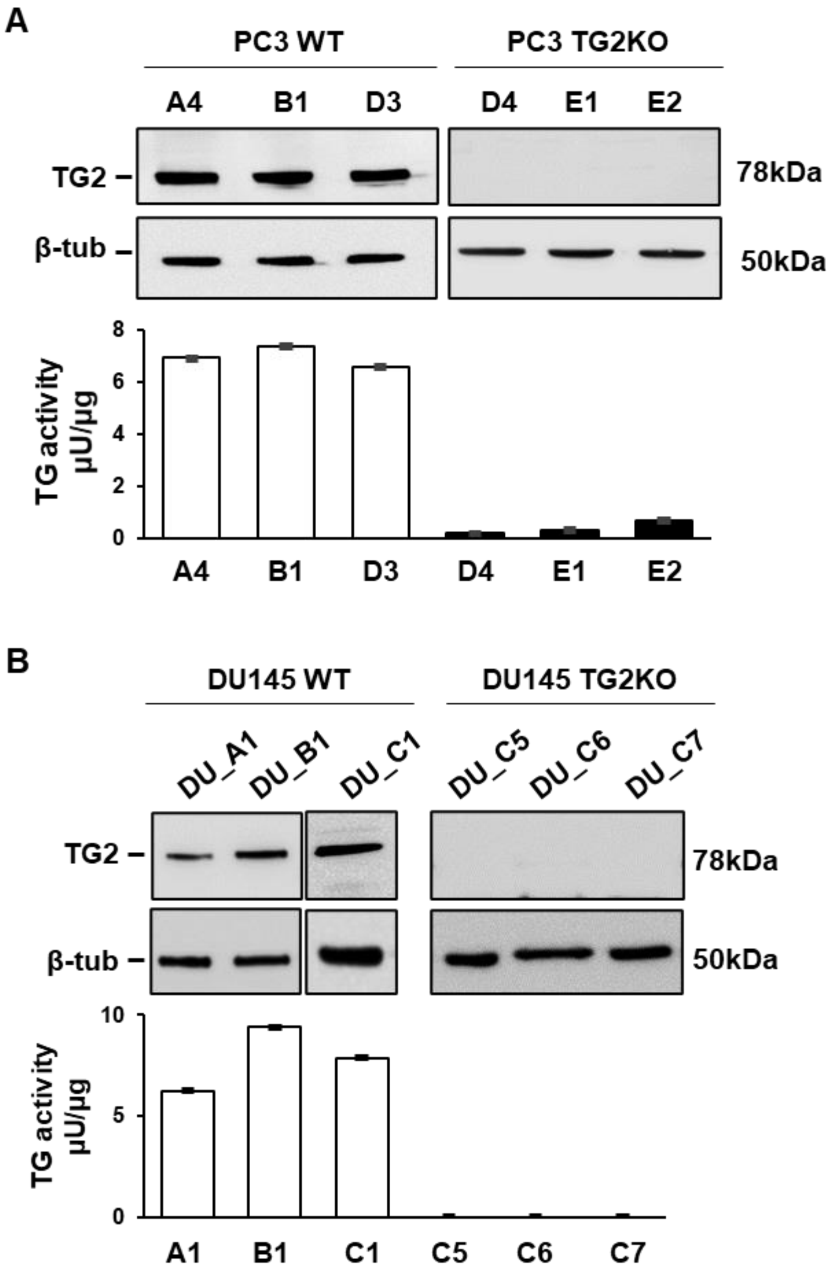

Supplementary Figure 5. Knockout of TGM2 in metastatic androgen insensitive PCa cell lines, PC3 and DU145 by CRISPR/Cas9. (**A, B**) Immunoblot showing expression of TG2 in six PC3 or six DU145 clones, following knockdown of TGM2 via CRISPR-Cas9. Proteins were separated by electrophoresis, immunoblotted and probed with either mouse monoclonal anti-transglutaminase-2 (CUB7402) or rabbit polyclonal anti-β-tubulin. Transamidating TG activity was measured in cell lysates by detecting the incorporation of biotinylated cadaverine into fibronectin as described in the supplementary methods. Total TG activity is represented in µU/µg.

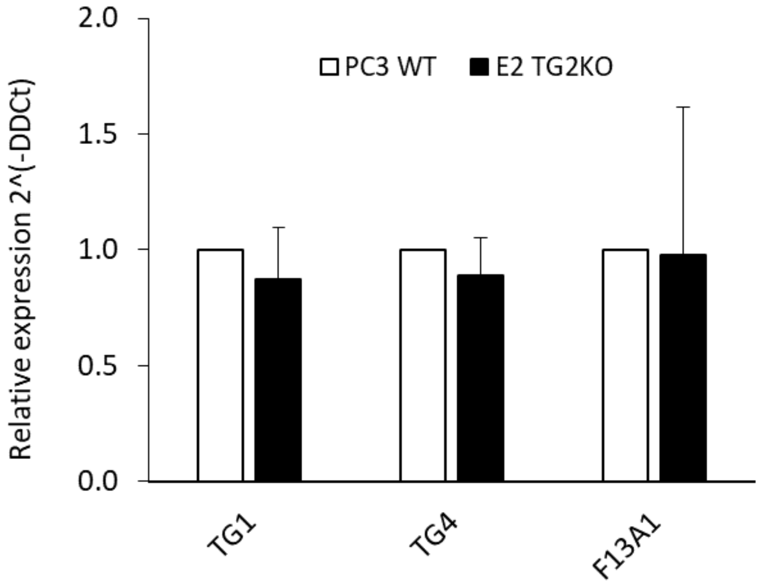

Supplementary Figure 6. Relative expression of main TG family members in TG2KO cells (E2 clone) relative to wild type PC3 cells. TG transcripts were amplified by qRT-PCR, using isoform-specific oligonucleotide-primers (Supplementary Table 6) and quantified by the $2^{-\Delta\Delta Ct}$ relative method as described in the Methods according to (2). The values were normalised to the mean expression of reference genes (HPRT1). Expression in WT cells was equal to 1 ($2^{-\Delta\Delta Ct}$ = 1). Data are the mean ± SEM of two independent experiments.

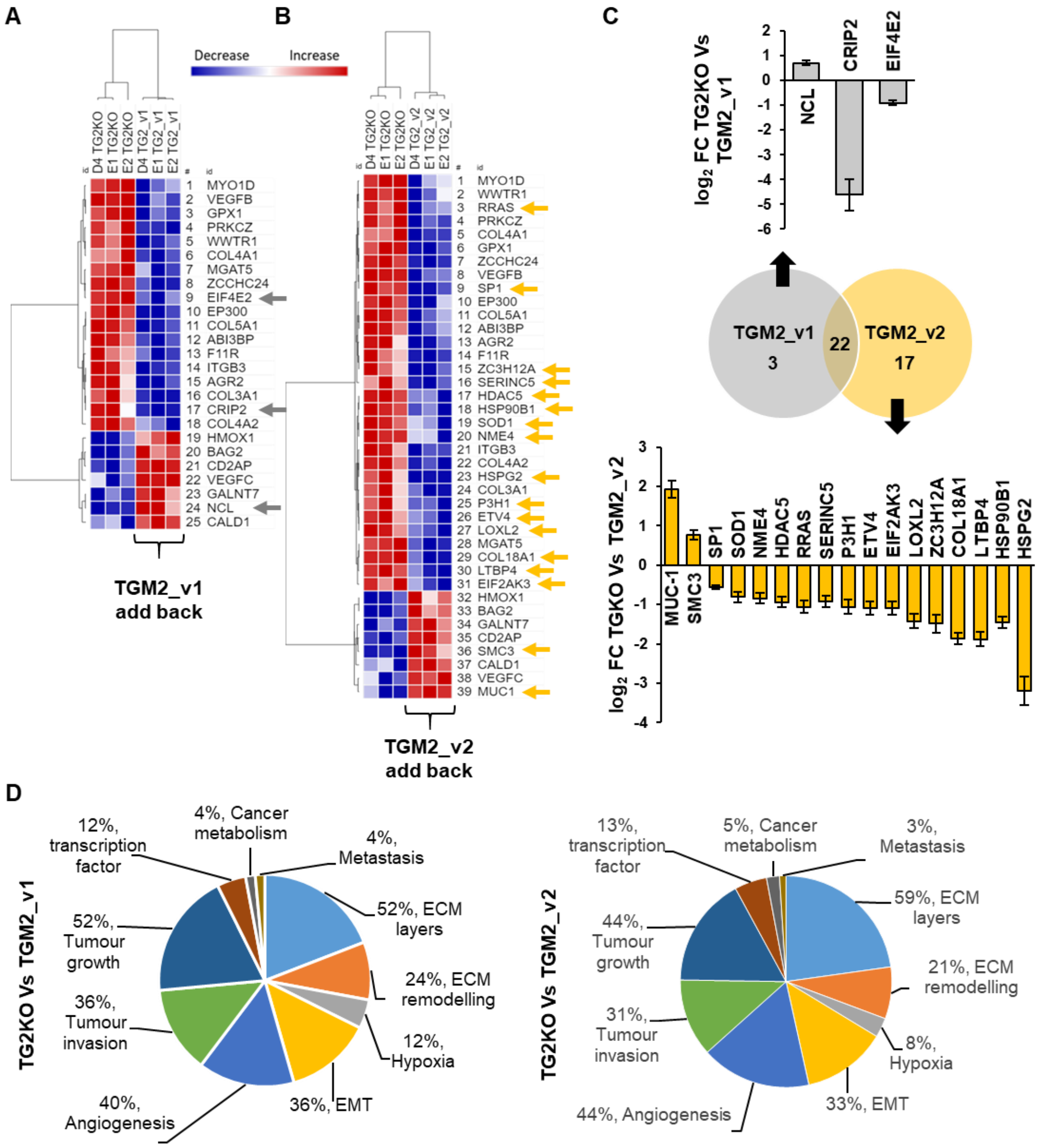

Supplementary Figure 7. Differentially expressed cancer-related genes induced by TGM2_v1 and/or TGM2_v2 and cancer processes implicated. (**A, B**) Heatmap displaying differentially expressed genes with its normalised mRNA log_2 count of each gene modulated by addback of TGM2_v1(A) and TGM2_v2 (B) using NanoString Cancer progression panel, data plotted using Morpheus from Broad Institute. Hierarchical clustering and One minus Pearson Correlation in rows and columns. Intense blue to less intense blue signifies range of gene underexpressed, while light red to intense red signifies range of gene overexpressed as indicated in the key. Grey arrows indicate genes that are uniquely modulated by TGM2_v1 and yellow arrows indicate genes uniquely modulated by TGM2_v2. (**C**) Venn diagram displaying common genes modulated by both TGM2 isoform, log_2 fold changes of these genes are

individually named in histograms. (**D**) Pie chart categorising the differentially expressed genes induced by either TGM2_v1 and TGM2_v2 into cancer progression categories according to NanoString Cancer progression panel. Each cancer progression category shown with the percentage of gene hit against total number of cancer progression categories.

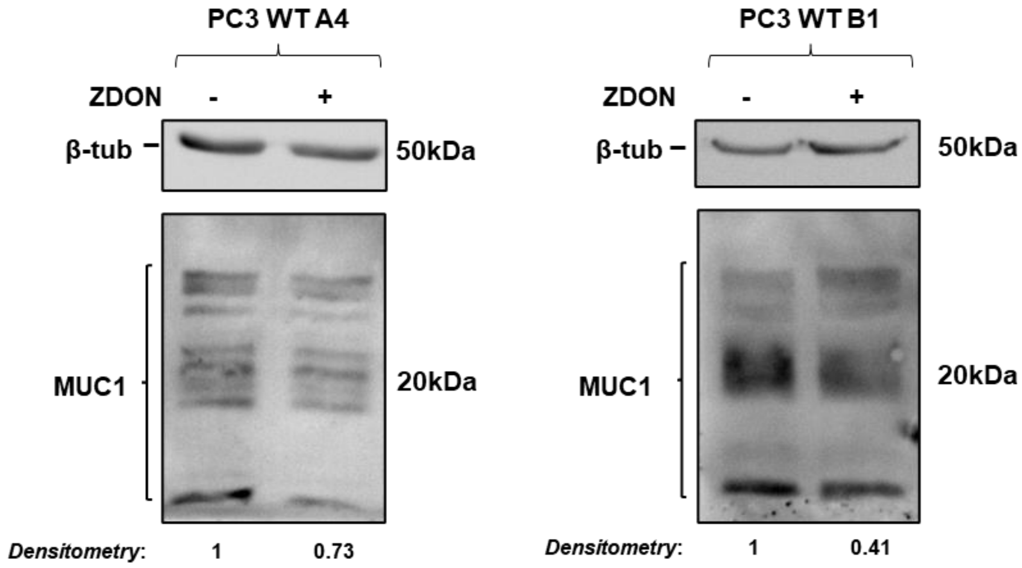

Supplementary Figure 8. MUC1 protein level is attenuated by pharmacological inhibition of TG2. PC3 WT cells (clones A4 and B1) were incubated with TG2 inhibitor ZDON (90 µM) for 24 hours, lysed in RIPA buffer and equal amounts (120 µg) analysed by western blotting in reducing conditions for detection of MUC1 and β-tubulin. Densitometry quantification of MUC1 bands normalised to β-tubulin and to untreated cells is shown. A typical experiment is shown.

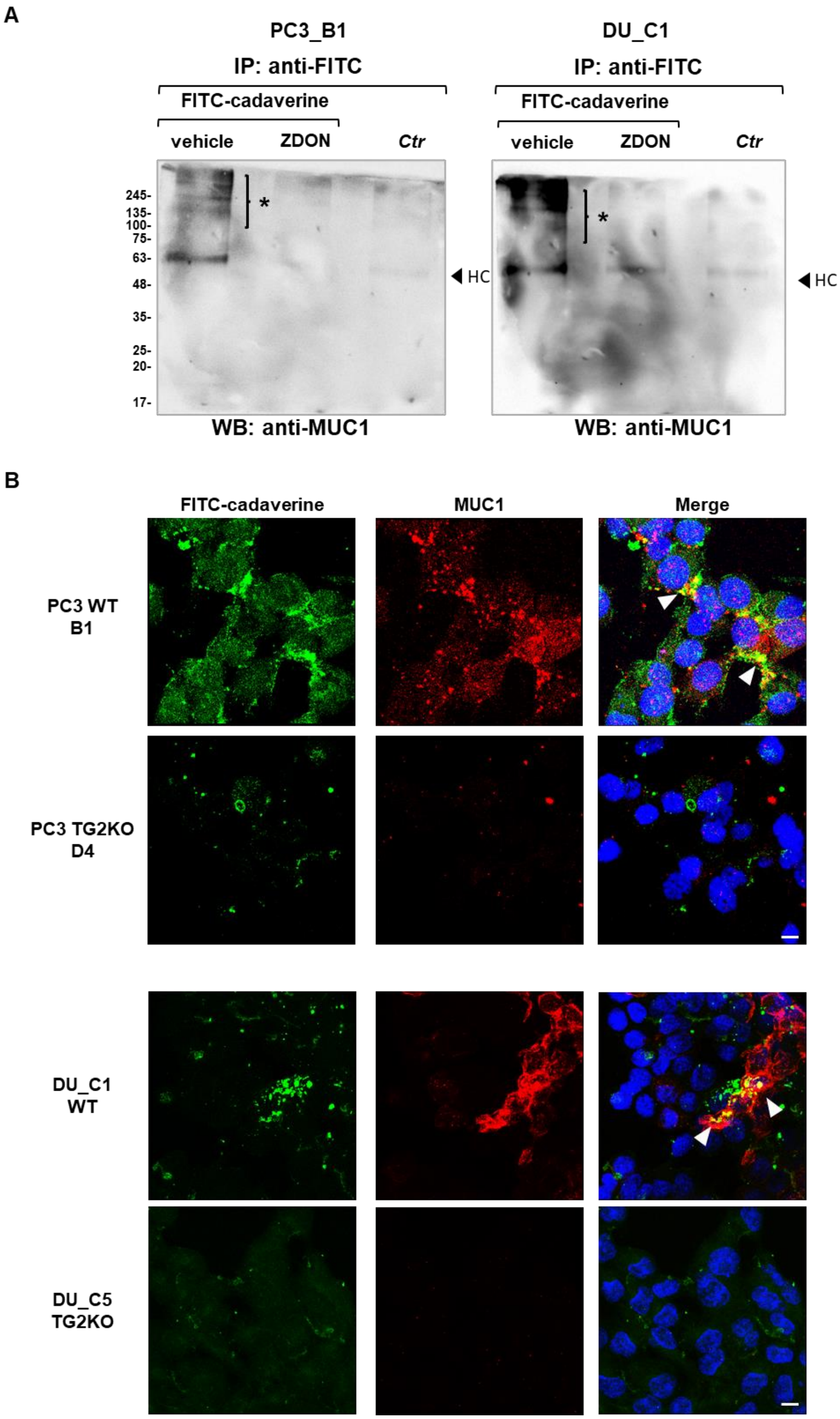

Supplementary Figure 9. MUC1 is a target of TG2-mediated crosslinking in PC3 and DU145 cells. A) PC3 or DU145 clonal cell lines were cultured in the presence of 0.5 mM

FITC-cadaverine for 5 h, lysed in the presence of EDTA and immunoprecipitated with mouse anti-FITC antibody (~230 μg of lysate) to recover substrates of transglutaminase as described in (3) and in the supplementary methods. TG2-mediated transamidation was inhibited by incubation with ZDON (90 μM). Eluted proteins were analysed by western blotting using anti MUC1 and secondary anti-rabbit-HRP antibody. One representative blot of two independent experiments per cell line, for a total of four experiments is shown. *: TG2-dependent MUC1 high molecular weight multimers. **B)** PC3 or DU145 clonal cell lines (WT and TG2KO) were cultured in the presence of 0.5 mM FITC-cadaverine for 15 h and fixed as previously described (4). Specimens were immunostained with anti-MUC1 antibody which was revealed by anti-rabbit AF-568 antibody. Nuclei were stained with DAPI. Specimens were imaged using fluorescence confocal microscopy (Leica SP5) using a 63X objective. Successive serial optical sections (1μm) were recorded over 8 μm planes and maximum projections are shown. The white arrowheads indicate points of co-localisation between in situ TG activity (green) and MUC1 protein (red). The degree of colocalization was evaluated by Leica co-localisation software. A typical experiment is shown. Scale bar: 10 μm.

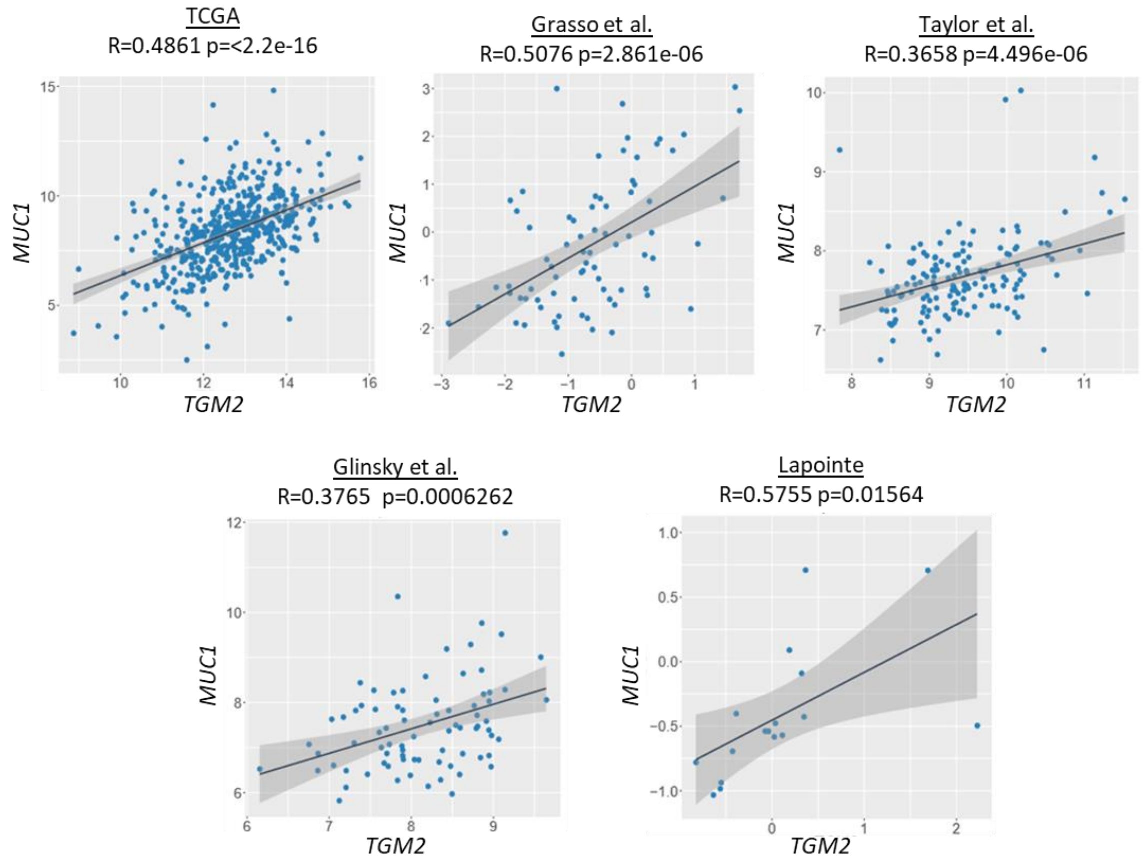

Supplementary Figure 10. TG2 expression correlates with MUC1 expression in prostate cancer patients. Correlation plot of TGM2 and MUC1 expression in primary and metastatic tumours from different prostate cancer databases. Size of samples for each study: Glinsky n =79, Grasso n =88, Taylor n =179, TCGA n =496 (https://www.cancer.gov/tcga), Lapointe n =26 (5–8). Black line represents linear regression, grey area indicates the limits of the confidence intervals and R and p indicate Pearson´s correlation coefficient and statistical significance, respectively.

Supplementary Tables

Supplementary Table 3. Significant differentially expressed genes in TG2KO cells (three TG2KO clones analysed) compared to WT cells (five WT clones analysed). Genes are labelled according to the HUGO gene nomenclature. Normal p-value subjected to the Benjamini-Hochberg (BH) correction test to exclude false discovery (BH p-value ≤ 0.05). Genes are listed in the order of increased expression levels (from least expressing to highest expression genes) in TG2KO cells when compared to WT cells.

Underexpressed genes					
HUGO Gene ID	Gene name	Log2 fold change	Linear fold change	P-value	BH.p.value
MUC-1	Mucin-1	-1.38	0.384	0.00131	0.0376
PLEKHO1	Pleckstrin homology domain containing, family O member 1	-0.847	0.556	3.02E-05	0.0148

Overexpressed genes					
HUGO Gene ID	Gene name	Log2 fold change	Linear fold change	P-value	BH.p.value
RPS6KB1	Ribosomal protein S6 kinase, 70kDa, polypeptide 1	0.429	1.35	0.000206	0.0187
SMURF2	SMAD specific E3 ubiquitin protein ligase 2	0.554	1.47	0.00206	0.0529
DLG1	Discs, large homolog 1	0.685	1.61	0.00188	0.0509
NF2	Neurofibromin 2	0.687	1.61	0.0024	0.0535
PDK1	Pyruvate dehydrogenase kinase, isozyme 1	0.707	1.63	0.000552	0.0208
ENO2	Enolase 2	0.888	1.85	0.00228	0.0535
PIK3CA	Phosphatidylinositol-4,5-bisphosphate 3-kinase, catalytic subunit alpha	0.908	1.88	0.000314	0.0192
PGK1	Phosphoglycerate kinase 1	0.939	1.92	0.000151	0.0186
BTG1	B-cell translocation gene 1	0.97	1.96	0.000152	0.0186
FGFR1	Fibroblast growth factor receptor 1	0.997	2	0.000495	0.0202
EIF4E2	Eukaryotic translation initiation factor 4E family member 2	1.05	2.08	0.000409	0.02
MMP14	Matrix metallopeptidase 14	1.16	2.23	0.000304	0.0192
ITGA3	Integrin, alpha 3	1.25	2.38	0.00023	0.0187
HSP90B1	Heat shock protein 90kDa beta (Grp94), member 1	1.34	2.54	0.00109	0.0354
MGAT5	Mannosyl (alpha-1,6-)-glycoprotein beta-1,6-N-acetyl-glucosaminyltransferase	1.48	2.79	0.00116	0.0354
ABI3BP	ABI family, member 3 (NESH) binding protein	1.55	2.93	0.000456	0.0202
ITGA5	Integrin, alpha 5	2	3.99	0.000979	0.0342
EPHB3	EPH receptor B3	2.06	4.17	8.30E-05	0.0186
ANGPTL4	Angiopoietin-like 4	2.22	4.67	0.000356	0.0194
COL6A3	Collagen, type VI, alpha 3	2.54	5.83	0.00235	0.0535

Supplementary Table 4. Differentially expressed genes in TGM2_v1 knock-in PC3 cells compared to TG2KO cells (three TG2KO clones analysed) with BH p-value ≤ 0.05. Normal p-value obtained was subjected to the Benjamini-Hochberg (BH) correction test to exclude false discovery rate (BH p-value ≤ 0.05). Genes are listed in the order of increased expression levels (from least expressing to highest expression genes) in TGM2_v1 cells when compared to TG2KO cells.

DIFFERENTIALLY EXPRESSED GENES IN TGM2_v1 Vs TG2KO						
	HUGO Gene ID	Gene name	Log2 fold change	Linear fold change	P-value	BH.p.value
Underexpressed genes	WWTR1	WW domain containing transcription regulator 1	-0.78	0.582	0.00109	0.0434
	PRKCZ	Protein kinase C, zeta	-0.829	0.563	0.000939	0.0434
	MYO1D	Myosin ID	-0.912	0.532	0.00247	0.0496
	EP300	E1A binding protein p300	-0.919	0.529	0.000109	0.0326
	VEGFB	Vascular endothelial growth factor B	-1.01	0.497	0.000688	0.0434
	GPX1	Glutathione peroxidase 1	-1.07	0.475	0.000629	0.0434
	COL5A1	Collagen, type V, alpha 1	-1.37	0.387	0.000291	0.0417
	F11R	F11 receptor	-1.37	0.386	0.00239	0.0496
	ABI3BP	ABI family, member 3 (NESH) binding protein	-1.41	0.378	0.000431	0.0417
	ZCCHC24	Zinc finger, CCHC domain containing 24	-1.8	0.288	0.000135	0.0326
	ITGB3	Integrin, beta 3	-1.86	0.275	0.00161	0.0493
	COL4A2	Collagen, type IV, alpha 2	-2	0.249	0.00185	0.0493
	COL4A1	Collagen, type IV, alpha 1	-2.08	0.236	0.00117	0.0434
	MGAT5	Mannosyl (alpha-1,6-)-glycoprotein beta-1,6-N-acetyl-glucosaminyltransferase	-2.29	0.205	0.00183	0.0493
	AGR2	Anterior gradient 2 homolog	-4.25	0.0526	0.00185	0.0493
	COL3A1	Collagen, type III, alpha 1	-6.47	0.0113	0.000834	0.0434
	CRIP2	Cysteine-rich protein 2	-4.62	0.0405	0.00194	0.0493
	EIF4E2	Eukaryotic translation initiation factor 4E family member 2	-0.903	0.535	0.00104	0.0434
Overexpressed genes	HMOX1	Heme Oxygenase 1	2.03	4.07	0.00256	0.0496
	BAG2	BCL2-associated athanogene 2	1.48	2.79	0.000882	0.0434
	GALNT7	UDP-N-acetyl-alpha-D-galactosamine:polypeptide N-acetylgalactosaminyltransferase 7 (GalNAc-T7)	1.29	2.45	0.00204	0.0493
	VEGFC	Vascular endothelial growth factor C	1.09	2.12	0.00184	0.0493
	CD2AP	CD2-associated protein	0.866	1.82	0.000424	0.0417
	CALD1	Caldesmon 1	0.862	1.82	0.00245	0.0496
	NCL	NCL- nucleolin	0.708	1.63	0.00251	0.0496

Supplementary Table 5. Differentially expressed genes in TGM2_v2 knock-in PC3 cells compared to TG2KO cells (three TG2KO clones analysed) with BH p-value ≤ 0.05. Normal p-value obtained was subjected to the Benjamini-Hochberg (BH) correction test to exclude false discovery rate (BH p-value ≤ 0.05). Genes are listed in the order of increased expression levels (from least expressing to highest expression genes) in TGM2_v2 cells when compared to TG2KO cells.

DIFFERENTIALLY EXPRESSED GENES IN TGM2_v2 Vs TG2KO						
	HUGO Gene ID	Gene Symbol and name	Log2 fold change	Linear fold change	P-value	BH.p.value
Underexpressed genes	SP1	Sp1 transcription factor	-0.567	0.675	0.000192	0.0239
	SOD1	Superoxide dismutase 1, soluble	-0.812	0.57	0.00389	0.0524
	NME4	NME/NM23 nucleoside diphosphate kinase 4	-0.84	0.559	0.00406	0.0533
	ZC3H12A	Zinc finger CCCH-type containing 12A	-1.49	0.357	0.00329	0.0498
	SERINC5	Serine incorporator 5	-0.921	0.528	0.00323	0.0498
	HDAC5	Histone deacetylase 5	-0.939	0.522	0.00186	0.0441
	LTBP4	Latent transforming growth factor beta binding protein 4	-1.88	0.272	0.00059	0.03
	COL18A1	Collagen, type XVIII, alpha 1	-1.87	0.273	0.000173	0.0239
	P3H1	Prolyl 3-hydroxylase 1 (P3H1)	-1.06	0.479	0.00374	0.0524
	RRAS	Related RAS viral (r-ras) oncogene homolog	-1.06	0.479	0.00271	0.0494
	ETV4	Ets variant 4	-1.09	0.47	0.00314	0.0498
	EIF2AK3	Eukaryotic translation initiation factor 2-alpha kinase 3	-1.1	0.468	0.00267	0.0494
	LOXL2	Lysyl oxidase-like 2	-1.42	0.374	0.00145	0.0402
	HSPG2	Heparan sulfate proteoglycan 2	-3.19	0.109	0.000981	0.035
	HSP90B1	Heat shock protein 90kDa beta (Grp94), member 1	-1.46	0.362	0.000443	0.03
	ABI3BP	ABI family, member 3 (NESH) binding protein	-1.47	0.36	0.00201	0.0457
	F11R	F11 receptor	-1.47	0.36	0.00141	0.0402
	ZCCHC24	Zinc finger, CCHC domain containing 24	-1.9	0.267	3.98E-05	0.0198
	ITGB3	Integrin, beta 3	-2.04	0.243	0.00174	0.0435
	COL4A2	Collagen, type IV, alpha 2	-2.21	0.216	0.000407	0.03
	COL4A1	Collagen, type IV, alpha 1	-2.23	0.213	0.000601	0.03
	VEGFB	Vascular endothelial growth factor B	-0.896	0.537	0.000538	0.03
	EP300	E1A binding protein p300	-0.967	0.512	0.0027	0.0494
	WWTR1	WW domain containing transcription regulator 1	-0.924	0.527	0.00381	0.0524
	COL5A1	Collagen, type V, alpha 1	-1.43	0.372	0.00137	0.0402
	GPX1	Glutathione peroxidase 1	-0.994	0.502	0.00025	0.025
	MGAT5	Mannosyl (alpha-1,6-)-glycoprotein beta-1,6-N-acetyl-glucosaminyltransferase	-2.48	0.179	0.00011	0.0239
	PRKCZ	Protein kinase C, zeta	-0.883	0.542	0.000804	0.035
	AGR2	Anterior gradient 2 homolog	-4.18	0.0552	0.00277	0.0494
	COL3A1	Collagen, type III, alpha 1	-5.7	0.0192	0.00254	0.0494
	MYO1D	Myosin ID	-0.858	0.552	0.00361	0.0524
Overexpressed genes	HMOX1	Heme Oxygenase 1	2.32	4.98	0.00305	0.0498
	BAG2	BCL2-associated athanogene 2	1.51	2.84	0.00112	0.0374
	GALNT7	UDP-N-acetyl-alpha-D-galactosamine:polypeptide N-acetylgalactosaminyltransferase 7 (GalNAc-T7)	1.22	2.33	0.00163	0.0428
	VEGFC	Vascular endothelial growth factor C	0.982	1.97	0.00314	0.0498
	CALD1	Caldesmon 1	0.862	1.82	0.00421	0.0539
	CD2AP	CD2-associated protein	0.773	1.71	0.000976	0.035
	SMC3	Structural maintenance of chromosomes 3	0.766	1.7	0.00237	0.0494
	MUC1	Mucin 1	1.92	3.78	0.000881	0.035

Supplementary Table 6. Primers used in this study.

Primer name	Primer sequence (5′ - 3′)	Product size(bp)
TGM2_v1 FW	CCTTACGGAGTCCAACCTCA	245
TGM2_v1 RV	CCGTCTTCTGCTCCTCAGTC	
TGM2_v2 FW	ACCGCTGAGGAGTACGTCTG	153
TGM2_v2 RV	TCAACAAATGCTCCAGGAA	
AR FW	TTGGATGGCTCCAAATCAC	148
AR RV	GCAATGATACGATCGAGTTCC	
HPRT FW	TGACACTGGCAAAACAATGCA	94
HPRT RV	GGTCCTTTTCACCAGCAAGCT	
TBP FW	TGCACAGGAGCCAAGAGTGAA	132
TBP RV	CACATCACAGCTCCCCACCA	
B-ACT FW	TTGTTACAGGAAGTCCCTTGCC	101
B-ACT RV	ATGCTATCACCTCCCCTGTGTG	
KRT13 FW	AGGACGCCAAGATGATTGGTT	70
KRT13 RV	GTGGTAACAGAGGTGCTACGG	
PCA3 FW	AGGTGAGAAATAAGAAAG	258
PCA3 RV	TAGAAACGAGTAGAGGA	
MUC1 FW	TACCGATCGTAGCCCCTATG	199
MUC1 RV	CTCACCAGCCCAAACAGG	
TGM1 FW	GGTGAACTCCCTGGATGACA	250
TGM1 RV	AAGGGATGTGTCTGTGTCGT	
TGM4 FW	AAGATGGTGAATGGGCAGGA	199
TGM4 RV	TGTGCTCCCTCTCAGAACTG	
F13A1 FW	TTGCTCAATACCTGGCCTCA	162
F13A1 RV	TGTTGTGCTCTAGTCCTGGG	

Supplementary Table 7. Tumour grade for patients recruited for gene expression analysis of TGM2_v1 and TGM2_v2.

**Biopsies from 57 patients accessed for gene expression study of TGM2 isoforms**		
**Type and number of patients recruited**	**Gleason score**	**Grade**
30 Chronic Idiopathic Prostatitis (CIP)	N/A	N/A
27 Prostate adenocarcinoma (PAC)	Gleason 3+3 & 3+4 (n=9)	1+2
	Gleason 4+3 (n=7)	3
	Gleason 4+4, 4+5 & 5+5 (n=11)	4+5

Supplementary Table 8. Analysis of off-target deletion likely to be introduced by Cas9 via computational analysis. List generated by computational analysis using the Zhang lab's tool. 20 nucleotide target sequences found similar to the sequence used to target exon 1 of TGM2 gene (CCCCGCCCGACCATGGCCGA) with possible mismatches on position (highlighted in red) of each sequence, corresponding scores and position of target sequence on the genomic locus.

Target sequence similar to sequence used to target TGM2

PAM sequence

sequence	score	mismatches	locus
CTTGGCCAGACCATGGCCGAAAG	1. 3	4MMs [2: 3: 4: 8]	chr 12: +111468149
CTCTGCCCGACCATGGCCTATGG	1. 1	3MMs [2: 4: 19]	chr 1: - 62625649
GCCTGCACGCCCATGGCCGAGAG	0. 9	4MMs [1: 4: 7: 10]	chr 15: +60681753
CCCACCACGCCCATGGCCGACAG	0. 9	4MMs [4: 5: 7: 10]	chr 6: - 159565264
CCCAGCCTGCACATGGCCGACGG	0. 7	4MMs [4: 8: 10: 11]	chr 9: - 128634486
CACCTCCCGTCCATGGCCGGTGG	0. 6	4MMs [2: 5: 10: 20]	chr 17: +73743911
AGCCGCTCTACCATGGCCGACAG	0. 6	4MMs [1: 2: 7: 9]	chr 5: +95745244
CGCCGCGACACCATGGCCGATGG	0. 6	4MMs [2: 7: 8: 9]	chr X: - 8169121
TCCCGCCTGGCCTTGGCCGATGG	0. 5	4MMs [1: 8: 10: 13]	chr 10: +50043750
CCCCTCCCTGCCATGGCCGTAAG	0. 4	4MMs [5: 9: 10: 20]	chr 10: +110783053
CCACACCCCACCCTGGCCGAAAG	0. 3	4MMs [3: 5: 9: 13]	chr 18: - 7458939
ACCCGCTCGCCCATGGCCAAAGG	0. 3	4MMs [1: 7: 10: 19]	chr 8: +69292791
CCCCACTCGCCCATGGCCTAGGG	0. 3	4MMs [5: 7: 10: 19]	chr 21: +45539423
CCCAACCCCACCATGGCCCACAG	0. 3	4MMs [4: 5: 9: 19]	chr 15: +28539453
CCCCTCCACACCATGGCCCAAAG	0. 3	4MMs [5: 8: 9: 19]	chr 10: +91464006
CCCCCACGGACCATGGCCAATGG	0. 3	4MMs [5: 6: 8: 19]	chr 21: +35526204
CCCAGCCCTCCCATGGCCCATGG	0. 3	4MMs [4: 9: 10: 19]	chr 7: +155080012
CCCCTCCCAGCCATGGCCTAGGG	0. 3	4MMs [5: 9: 10: 19]	chr 8: - 130531368
TCCTGCCCGACCCTGGCCGGGAG	0. 3	4MMs [1: 4: 13: 20]	chr 17: +45981074
CGCCGCCTGACCCTGGCCGCAGG	0. 3	4MMs [2: 8: 13: 20]	chr 1: - 43505168
GCCCGCCCGCCGCTGGCCGACAG	0. 3	4MMs [1: 10: 12: 13]	chr 6: - 153505101
CCCAGCCCGCCCTTGGCCGTAAG	0. 2	4MMs [4: 10: 13: 20]	chr 1: - 193533759
CTCCGCCTGACCATGGCCTGCAG	0. 2	4MMs [2: 8: 19: 20]	chr 1: - 62382764
CCCCGACCCACCATGGCAGAGAG	0. 2	3MMs [6: 9: 18]	chr 8: - 53852890
CCCCGGGCGCCCCTGGCCGATAG	0. 2	4MMs [6: 7: 10: 13]	chr X: - 9293475
CCCCGAGGGACCATGGCCAAAGG	0. 2	4MMs [6: 7: 8: 19]	chr 16: - 427311
CCTCGCCTGACCTTGGCCCAAAG	0. 2	4MMs [3: 8: 13: 19]	chr 5: +136408413
CACAGCCCTACCATGGCTGATGG	0. 2	4MMs [2: 4: 9: 18]	chr 19: +36099793
CCCCTCCAGACCCTGGCCAAAGG	0. 2	4MMs [5: 8: 13: 19]	chr 19: +56338748
CCCCACCCCAGCCTGGCCGAAAG	0. 2	4MMs [5: 9: 11: 13]	chr X: +16247885
CCCAGGCAGACCATGGCAGACAG	0. 2	4MMs [4: 6: 8: 18]	chr 3: - 129967548
CCCAGTCAGACCATGGCGGAAGG	0. 2	4MMs [4: 6: 8: 18]	chr 3: +129953809
CCCGGGCCTACCATGGCCCACAG	0. 2	4MMs [4: 6: 9: 19]	chr 5: +117090159
CCCGGCCCCAGCATGGCCCATAG	0. 2	4MMs [4: 9: 11: 19]	chr 6: - 14980560
CCCCGCCTGCCCATGGACCATGG	0. 2	4MMs [8: 10: 17: 19]	chr 20: - 51798649
CCCCGACCTTCCATGGCCCACAG	0. 2	4MMs [6: 9: 10: 19]	chr X: - 129195459
CCCCGGCGGACGGTGGCCGAGGG	0. 2	4MMs [6: 8: 12: 13]	chr 20: - 11603806
CCCGACACGACCAAGGCCGAGAG	0. 1	4MMs [4: 5: 7: 14]	chr 8: - 132370185
CTCAGCCCAACCAAGGCCGAGGG	0. 1	4MMs [2: 4: 9: 14]	chr 12: - 127108506
CCCCGCCCTGCCGTGGCCGGAGG	0. 1	4MMs [9: 10: 13: 20]	chr 7: +130403085
CCGCGTCCGAGCATCGCCGACAG	0. 1	4MMs [3: 6: 11: 15]	chr 21: - 38495057
CCCTGCACGACCCTGGCCCAGGG	0. 1	4MMs [4: 7: 13: 19]	chr 2: - 216536205
CCCTCCCCGACCATGGCTGCTAG	0. 1	4MMs [4: 5: 18: 20]	chr 5: +9702729
CCTCGCCCCACCATGGCCACTAG	0. 1	4MMs [3: 9: 19: 20]	chr 21: - 38261573
CCCCTCCAGACCATGGCTGTAGG	0. 1	4MMs [5: 8: 18: 20]	chr 11: +47389410
CCCCTCCCCACCATGGCCCGTAG	0. 1	4MMs [5: 9: 19: 20]	chr 6: - 71999834
CCCCGCCCCAGCAAGGCCGATGG	0. 1	3MMs [9: 11: 14]	chr 3: +100104277
TCCCGCCCCACCATGGGCAATGG	0. 1	4MMs [1: 9: 17: 19]	chr 8: - 98290785
CCCAGCCAGACCCTGGCAGATAG	0. 1	4MMs [4: 8: 13: 18]	chr 18: +13104013

Supplementary Table 9. Differential regulation of genes in TG2KO PC3 cells compared to WT PC3 cells with a Benjamini-Hochberg p-value with trend toward significance. Normal p-value was subjected to the (BH) correction test to exclude false discovery rate (BH p value of genes listed below fall between 0.0575 – 0.1).

Underexpressed genes - trend towards significance					
HUGO Gene ID	Gene name	Log2 fold change	Linear fold change	P-value	BH.p.value
SLPI	Secretory leukocyte peptidase inhibitor	-2.43	0.185	0.00286	0.0575
BMP4	Bone morphogenetic protein 4	-1.69	0.309	0.00421	0.0606
SMC3	Structural maintenance of chromosomes 3	-0.451	0.175	0.00692	0.0705
NOX5	NADPH oxidase, EF-hand calcium binding domain 5	-2.51	0.142	0.01	0.0907
EGLN2	Egl nine homolog 2	-0.367	0.71	0.00997	0.0907
DESI1	Desumoylating isopeptidase 1	-0.495	0.731	0.0122	0.0966
SMAD9	SMAD family member 9	-2.81	0.775	0.0138	0.1

Overexpressed genes- trend towards significance					
HUGO Gene ID	Gene name	Log2 fold change	Linear fold change	P-value	BH.p.value
SOD1	Superoxide dismutase 1, soluble	0.47	1.39	0.00324	0.0575
CHP1	Calcineurin-like EF hand protein 1	0.516	1.43	0.00308	0.0575
WWTR1	WW domain containing transcription regulator 1	0.737	1.67	0.00299	0.0575
TCF4	Transcription factor 4	1.4	2.64	0.00329	0.0575
GPR56	G protein-coupled receptor 56	2.12	4.34	0.00305	0.0575
ETV4	Ets variant 4	0.632	1.55	0.00403	0.0606
LAMB3	Laminin, beta 3	1.79	3.45	0.0038	0.0606
IL1B	Interleukin 1, beta	1.79	3.46	0.00419	0.0606
SNAI2	Snail homolog 2	1.27	2.41	0.00507	0.0653
ALDOA	Aldolase A, fructose-bisphosphate	0.443	1.36	0.00628	0.0682
VIM	Vimentin	0.663	1.58	0.00561	0.0682
TGFB1	Transforming growth factor, beta 1	0.667	1.59	0.00582	0.0682
EP300	E1A binding protein p300	0.886	1.85	0.00552	0.0682
SLC2A1	Solute carrier family 2 (facilitated glucose transporter), member 1	1.08	2.11	0.00614	0.0682
LAMA5	Laminin, alpha 5	1.04	2.06	0.00645	0.0685
ID2	Inhibitor of DNA binding 2, dominant negative helix-loop-helix protein	1.96	3.88	0.00726	0.0725
MAP2K2	mMitogen-activated protein kinase kinase 2	0.459	1.37	0.00764	0.0747
PTK2	Protein tyrosine kinase 2	0.66	1.58	0.00902	0.0864
BMPR2	Bone morphogenetic protein receptor, type II (serine/threonine kinase)	0.595	1.51	0.00961	0.0904
SP1	Sp1 transcription factor	0.538	1.45	0.0102	0.0909
JUN	Jun proto-oncogene	0.938	1.92	0.0104	0.0909
FGFR4	fibroblast growth factor receptor 4	0.972	1.96	0.0117	0.0956
ROCK1	Rho-associated, coiled-coil containing protein kinase 1	0.352	1.28	0.0122	0.0966
F11R	F11 receptor	1.06	2.08	0.0125	0.0967
LOXL2	Lysyl oxidase-like 2	1.21	2.32	0.0127	0.0967
TWIST1	Twist homolog 1	0.801	1.74	0.0131	0.0973
VEGFB	Vascular endothelial growth factor B	0.9	1.87	0.0131	0.0973
FN1	Fibronectin 1	2.78	6.87	0.0162	0.109

Supplementary Methods

Cell culture conditions

Cells were cultured in humidified culture conditions with 5% CO_2, 37°C. All cell lines used were tested for negative mycoplasma infection using EZ-PCR Mycoplasma test (Biological Industries, Israel). PCa cell lines were used between passage 10-40. Hypoxia was induced by modular incubator hypoxia chamber (Billups-Rothenberg Inc, San Diego, CA, USA) or chemically induced applying $CoCl_2$ (100-150 µM). PC3 cells were cultured in DMEM F-12 (Dulbecco's Modified Eagle Medium: Nutrient Mixture F-12) supplemented with 10% FBS (BioWest, Nuaillé, France) and 1% Penicillin-Streptomycin. DU145 cells in EMEM supplemented with 10% FBS, 1% Penicillin-Streptomycin, 1% Non-essential amino acids. LNCaP in RPMI-1640 with 10% FBS, 1% Penicillin-Streptomycin and 1% Sodium pyruvate. PC346-Flu1 and PC346-Flu2 were cultured in DMEM-F12 supplemented with 0.001% BSA (Sigma-Aldrich, St. Louis, MO, USA), 2% DCC, 10 ng/mL EGF (Sigma-Aldrich), 1% Insulin-Transferrin-Selenium (Gibco, Waltham, MA, USA), 0.5 µg/mL hydrocortisone (Sigma-Aldrich), 0.6 ng/mL triiodothyronine (Sigma-Aldrich), 0.1 mM phosphoethanolamine (Sigma-Aldrich), 50 ng/mL cholera toxin (Sigma-Aldrich), 100 ng/mL fibronectin (Sigma-Aldrich), 20 µg/mL Fetuin (ICN Biomedicals Inc., Costa Mesa, CA, USA) and 1µM hydroxyflutamide (Sigma-Aldrich). All growth media were commercially obtained from Lonza (Manchester, UK).

Quantitative PCR (qRT-PCR)

Total RNA was isolated using the GenElute™ Mammalian total RNA miniprep kit (Sigma-Aldrich) and reverse transcribed (2µg) with random primers (Promega UK, Chilworth, UK) using Superscript® II Reverse Transcriptase (Invitrogen, Waltham, MA, USA). cDNA was amplified by qPCR using iQSYBR Green Supermix (Bio-Rad, Hercules, CA, USA), with oligonucleotide primer pairs listed in Supplementary Table 6, as previously described (9). Relative quantifications were performed by the $2^{-\Delta\Delta Ct}$ according to (2) with HPRT-1 or TBP as housekeeping gene.

Screening of TG2 transcripts in patients' biopsies

Male patients (mean age: 65.1 ± 6.3 years), who had undergone transrectal ultrasound–guided needle biopsy at the Urology Department of the Policlinic University Hospital (Messina, Italy)

were recruited for this study (n=101). Prostate cancer biopsy tissues were collected, processed, and classified (for classification see Supplementary Table 7) by Marta Rossanese as previously described (10). The study was approved by the Research Ethics Committee of Polyclinic Hospital University in Messina (approval number 37/17, date 2017/05/10), and carried out in accordance with Helsinki declaration ethical principles. Informed written consent was obtained from all participants.

Total RNA was isolated from prostate biopsies using the TRIzol reagent (ThermoFisher Scientific, Monza, Italy), and reverse transcribed using High-Capacity cDNA Archive Kit (ThermoFisher Scientific). TGM2_v1, TGM2_v2, Prostate Cancer Antigen (PCA3), and Keratin 13 (KRT13) transcripts, the latter two as markers of tumour and epithelial cells, respectively (10,11), were amplified (Supplementary Table 6) by SYBR Green-based Real-time qRT-PCR. PCA3− biopsies were excluded from the PRAD group as not representative of tumour samples, in order to decrease the chances of false positives. Similarly, PCA3+ biopsies were excluded from CIP group as representative of benign prostatic hyperplasia (BPH) instead of CIP. Power analysis was performed to determine the sample size using previously published data (power 80%, alpha 0.05) (6).

Real-Time qRT-PCR was performed in a 7900HT Fast Real-Time PCR System and data were collected with SDS 2.3 software (Applied Biosystems, Foster City, CA). The $2^{-\Delta Ct}$ method was used for assessing the levels of mRNA transcripts. β-actin (B-ACT) was used as housekeeping gene. The respective amounts of PCA3 $2^{-\Delta Ct}$ were used to normalise the expression levels of TGM2_v1 and TGM2_v2, calculated as $2^{-\Delta Ct}$, in PRAD samples (11). Differential expression levels were then expressed as fold change of normalised $2^{-\Delta Ct\ TGM2}$ values of PRAD compared to the average $2^{-\Delta Ct\ TGM2}$ of all CIP samples.

Immunofluorescence staining

TG2 immunostaining was performed, and specimens viewed by confocal microscopy as previously described (12), with the exception that cells were fixed and permeabilised 90% methanol in PBS (v/v) at −20°C for 10 min.

Immunoblotting of cell lysates

Cell lysates were prepared in sucrose lysis buffer (0.25M sucrose, 2mM EDTA and 5mM Tris-HCl) as previously described in(12). Equal protein amount was resolved by SDS-PAGE, 12% (w/v) acrylamide reducing and denaturing conditions, blotted using standard procedures and detected via primary antibody and secondary HRP-conjugated antibody by enhanced chemiluminescence (EZ-chemiluminescent Kit, Biological Industries) as previously described (13).

Cell proliferation, migration and adhesion

PCa cells ($3x10^3$ cells/well of 96well plate) were transferred into the IncuCyte S3 live cell imaging system for image acquisition every 2 hours (Essen Bioscience–Sartorius UK, Epsen, UK). Proliferation was expressed as percentage of phase area confluence normalised to time 0h. Migration was monitored by a scratch assay in real time PCa cells (40,000 cells/well) were seeded in wells of ImageLock 96well plate (Essen Bioscience) and allowed to form a monolayer. A 96 pin WoundMaker® was used to create a wound in the monolayer of cells. followed by image acquisition every 2 hours. Migration was expressed as relative wound density, i.e. the ratio of the occupied area to the total area of the original scratch area. Cell adhesion on fibronectin was performed as described (4) in serum starved cells (growth media containing 0.1% FBS).

Measurement of TG activity

Total TG activity was measured through incorporation of biotinylated cadaverine into fibronectin as previously described (14). In situ TG activity was measured through incorporation of FITC-cadaverine (0.5 mM) in live cells as previously described (4).

Expression constructs and cell transfections

PCa cells were transfected by electroporation using the Nucleofector® Kit V (Lonza) following manufacturer's instruction. pcDNA3.1(+)ValTG2 was a kind gift from Prof Fesus Laszlo and Róbert Király, University of Debrecen. Recombinant human TGM2_v1 was expressed and purified as previously described (1); the cDNA of recombinant human TGM2_v2 was subcloned from the Gateway PLUS shuttle GC-Z2235 (GeneCopoeia) into the pET21a(+)

plasmid (Novagen) between *NheI* and *HindIII* restriction enzyme sites. TGM2_v2 protein expression was induced in BL21 (DE3) cells (Novagen) as described (1). TGM2_v2 was also subcloned in pcDNA3.1/Hygro(-) via the *Nhel* and *HindIII* restriction enzyme site (pcDNA3.1/Hygro(-)TGM2_v2). Lipofectamine 3000 (Invitrogen) was used for the siRNA knockdown targeting the Androgen Receptor.

Antibodies and Reagents

Primary antibodies: mouse monoclonal anti-TG2 (CUB7402) (MA5-12739, Invitrogen); rabbit polyclonal anti-β-tubulin (Ab6046), mouse monoclonal anti CD63 (ab193349) and rabbit monoclonal anti-MUC1 (ab109185) (Abcam, Cambridge, UK); mouse monoclonal anti HIF-1α (MAB1536, Bio-Techne, UK); rabbit polyclonal anti-ALIX (pab0204, Covalab, France); mouse monoclonal anti Flotillin-2 (BD Biosciences, Wokingham, UK); mouse monoclonal anti-TOM20 (Santa Cruz Biotechnology, Dallas, TX, USA). Custom made rabbit antibody anti-TGM2_v2 was raised against the unique epitope $SG^{539}KALCSWSIC^{548}$ (exon 10b) (9); the immunogen was the N-terminal acetylated peptide in conjugation with KLH (Keyhole Limpet Hemocyanin) (GenScript, USA). Secondary antibodies: polyclonal IgG-HRP conjugated antibodies (Dako, Denmark); mouse IgG FITC conjugated antibodies (Sigma-Aldrich). Reagents: purified guinea pig TG2 (Sigma-Aldrich); G418 (Gibco); electroporation transfection kit V (Lonza); ZDON (Zedira, Darmstadt, Germany) used at IC_{50} 90µM; siRNA directed towards the androgen receptor (ThermoFisher Scientific, UK).

Immunoprecipitations

Immunoprecipitations from cell lysates were carried out by using the Pierce Crosslink Magnetic IP/Co-IP Kit (Thermo Scientific) as previously described (3).

Genotyping approach

Genomic DNA was extracted from the transfected cell lines by using the Qamp DNA mini kit (Qiagen, UK) according to manufacturers' instructions. The gene editing events in TGM2 exon 1 leading to reading frame disruption were detected by Sanger sequencing of PCR products amplified with primers flanking the targeted exon fragment, with the following primer pair:

Forward 5’-CCAGTGGTCGCACTTGGAGGG-3’
Reverse 5’-CCTGGCGGACCACTGGCACGT -3’

Spheroid Formation Assay

DU145 WT and TG2KO clones were cultured to ~80% confluency in T75 flasks, collected, counted and seeded into an ultra-low attachment 96-well plate (BRAND, USA) at 4,000 cells/well seeding density in complete EMEM medium. The plate was then centrifuged at 130 x g for 15 minutes and inserted into the IncuCyte® S3 live-cell analysis system. Images were taken at 6-hour intervals at 10x magnification for 16 days. Throughout the experimental duration no media change occurred.

Nuclear fractionation

Nuclear fractionation of PC3 cells was performed according to manufacturer’s instructions using NE-PER Nuclear and Cytoplasmic Extraction Reagents (Thermo Scientifc).

Prostate tissue microarray immunostaining

A FFPE prostate cancer tissue microarray (TMA, TissueArray.com) was deparaffinized using a series of washes, including three washes of 5 min each in xylene (534056, Sigma-Aldrich), followed by two washes in 100% ethanol (1009832511, Sigma-Aldrich), and two washes in 95% ethanol. The slide was then rehydrated in double distilled water and immediately subjected to antigen retrieval using 1X citrate buffer (C9999, Sigma-Aldrich) in a pre-heated pressure cooker for 15 min. The TMA was washed with 1X tris-buffered saline containing 0.1% Tween20 (TBS-T) for 5 min and blocked using 200µL of Buffer-W (11238417232, NanoString) for 1 h at room temperature in a humidity chamber. Mouse anti-TG2 (CUB7402, 1:100) and rabbit anti-TGM2_v2 (1:100) antibodies were then added to the tissues and incubated for 1 h at room temperature in a humidity chamber, followed by three washes (10 min each) with 1X TBS-T. The secondary antibodies (Alexa Fluor® 647 Goat Anti Mouse IgG H&L, 1:100, Abcam; Alexa Fluor® 594 Goat Anti rabbit IgG H&L, 1:100, Abcam) were prepared in Buffer-W and incubated in a humidity chamber protected from light for 1 h. The slide was washed three times (10 min each) with 1X TBS-T and nuclear staining of the tissues was performed with Syto13 (400nM, ThermoFisher Scientific) for 15 min at room temperature.

Excess Syto13 stain was removed with one 1X TBS wash and the TMA imaged using the NanoString GeoMx Digital Spatial profiling platform. Fluorescence intensities were measured with ImageJ Fiji software. Normal Adjacent Tissues (NAT) presenting cystic dilatation were exclude from the analysis.

Supplementary References

1. Lortat-Jacob H, Burhan I, Scarpellini A, Thomas A, Imberty A, Vivès RR, et al. Transglutaminase-2 Interaction with Heparin. Journal of Biological Chemistry. 2012 May;287(22):18005–17.

2. Livak KJ, Schmittgen TD. Analysis of Relative Gene Expression Data Using Real-Time Quantitative PCR and the 2−ΔΔCT Method. Methods. 2001 Dec;25(4):402–8.

3. Burhan I, Furini G, Lortat-Jacob H, Atobatele AG, Scarpellini A, Schroeder N, et al. Interplay between transglutaminases and heparan sulphate in progressive renal scarring. Sci Rep. 2016 Oct 3;6(1):31343.

4. Scarpellini A, Germack R, Lortat-Jacob H, Muramatsu T, Billett E, Johnson T, et al. Heparan Sulfate Proteoglycans Are Receptors for the Cell-surface Trafficking and Biological Activity of Transglutaminase-2. Journal of Biological Chemistry. 2009 Jul;284(27):18411–23.

5. Glinsky G V., Glinskii AB, Stephenson AJ, Hoffman RM, Gerald WL. Gene expression profiling predicts clinical outcome of prostate cancer. Journal of Clinical Investigation. 2004 Mar 15;113(6):913–23.

6. Grasso CS, Wu YM, Robinson DR, Cao X, Dhanasekaran SM, Khan AP, et al. The mutational landscape of lethal castration-resistant prostate cancer. Nature. 2012 Jul 20;487(7406):239–43.

7. Taylor BS, Schultz N, Hieronymus H, Gopalan A, Xiao Y, Carver BS, et al. Integrative Genomic Profiling of Human Prostate Cancer. Cancer Cell. 2010 Jul;18(1):11–22.

8. Lapointe J, Li C, Higgins JP, van de Rijn M, Bair E, Montgomery K, et al. Gene expression profiling identifies clinically relevant subtypes of prostate cancer. Proceedings of the National Academy of Sciences. 2004 Jan 20;101(3):811–6.

9. Phatak VM, Croft SM, Rameshaiah Setty SG, Scarpellini A, Hughes DC, Rees R, et al. Expression of transglutaminase-2 isoforms in normal human tissues and cancer cell lines: dysregulation of alternative splicing in cancer. Amino Acids. 2013 Jan 17;44(1):33–44.

10. Guttilla A, Zazzara M, Zattoni F, Novara G, Zanin M, Gardiman M, et al. Histopathological characteristics of microfocal prostate cancer detected during systematic prostate biopsy. BJU Int. 2015 Aug;116(2):202–6.

11. Cannata A, De Luca C, Korkina LG, Ferlazzo N, Ientile R, Currò M, et al. The SNP rs2298383 Reduces ADORA2A Gene Transcription and Positively Associates with Cytokine Production by Peripheral Blood Mononuclear Cells in Patients with Multiple Chemical Sensitivity. Int J Mol Sci. 2020 Mar 9;21(5):1858.

12. Furini G, Schroeder N, Huang L, Boocock D, Scarpellini A, Coveney C, et al. Proteomic Profiling Reveals the Transglutaminase-2 Externalization Pathway in Kidneys after

Unilateral Ureteric Obstruction. Journal of the American Society of Nephrology. 2018 Mar;29(3):880–905.

13. Tonoli E, Verduci I, Gabrielli M, Prada I, Forcaia G, Coveney C, et al. Extracellular transglutaminase-2, nude or associated with astrocytic extracellular vesicles, modulates neuronal calcium homeostasis. Prog Neurobiol. 2022 Sep;216:102313.

14. Jones RA, Nicholas B, Mian S, Davies PJ, Griffin M. Reduced expression of tissue transglutaminase in a human endothelial cell line leads to changes in cell spreading, cell adhesion and reduced polymerisation of fibronectin. J Cell Sci. 1997 Oct 1;110(19):2461–72.
