# Supplementary material for: Canonical and truncated transglutaminase-2 regulate mucin-1 expression and androgen independency in prostate cancer cell lines
Source: Cell Death Dis. 2023 May 9;14(5):317. doi: 10.1038/s41419-023-05818-9 (PMC10170068; doi:10.1038/s41419-023-05818-9)

Figure 1A

(A)

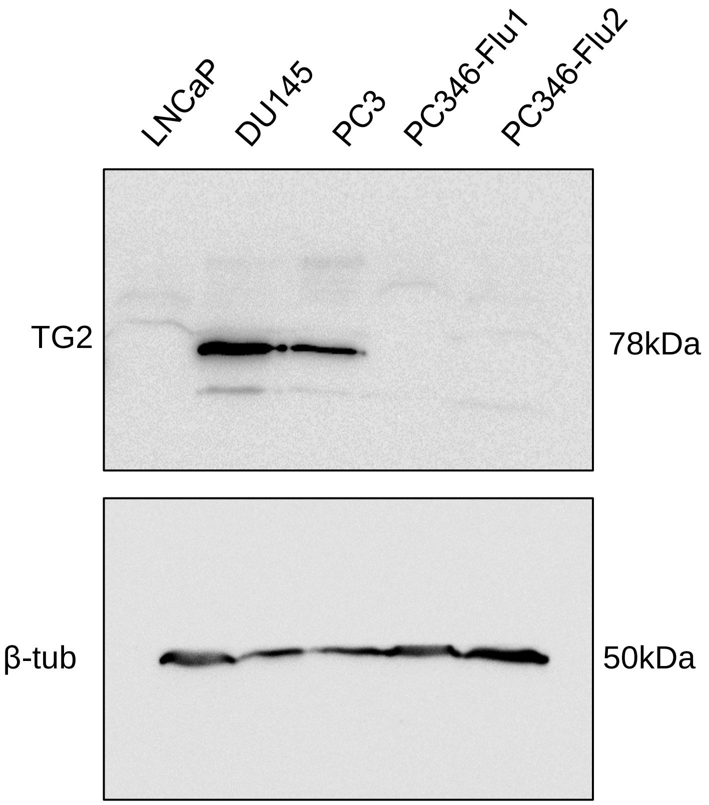

Figure 1B

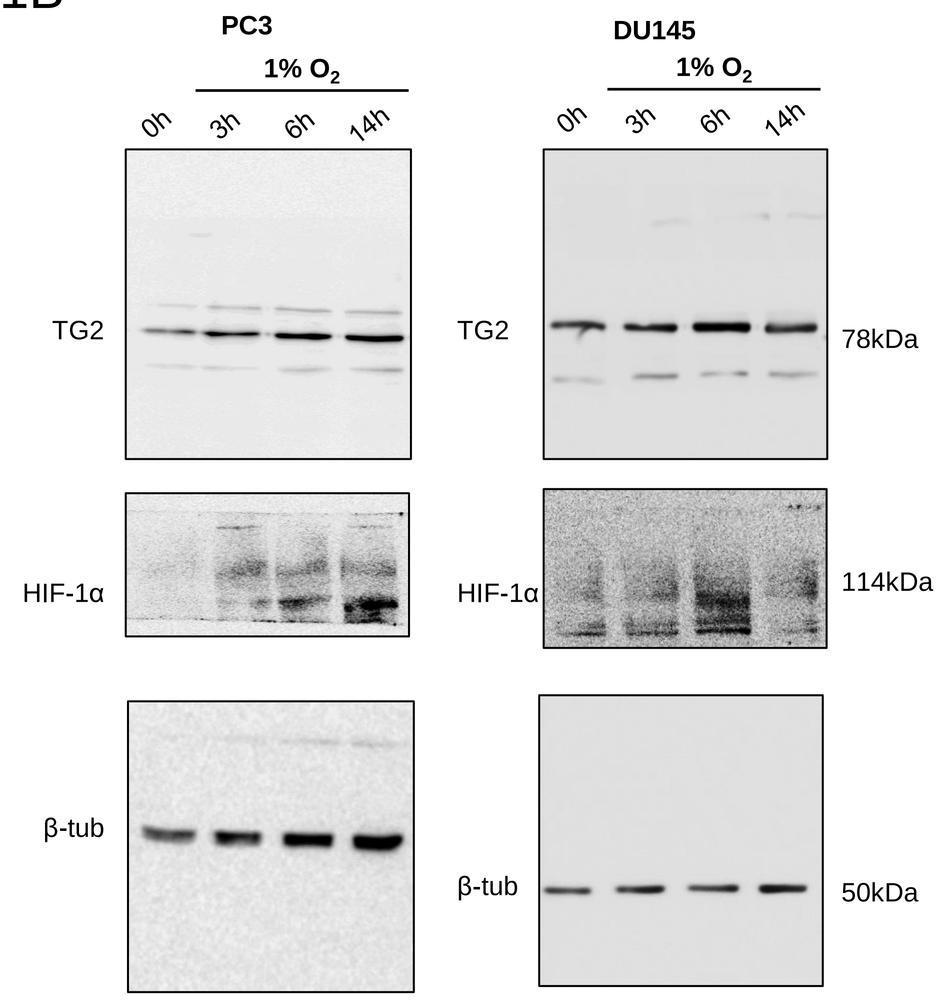
PC3
1% O_2
0h
3h
6h
14h
TG2
HIF-1α
β-tub
DU145
1% O_2
0h
3h
6h
14h
TG2
HIF-1α
β-tub
78kDa
114kDa
50kDa

Figure 1C

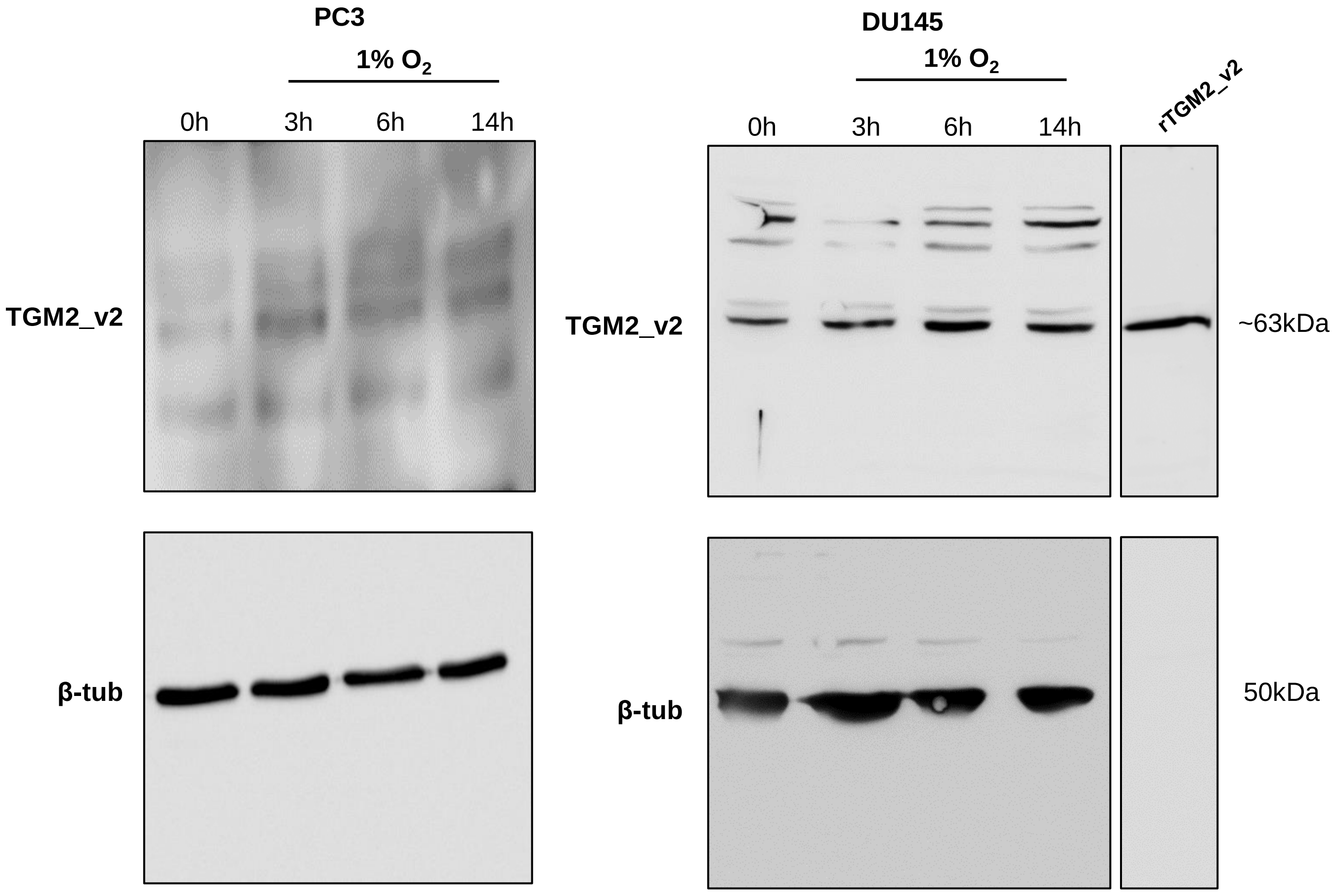
PC3
1% O$_2$
0h
3h
6h
14h
TGM2_v2
β-tub
DU145
1% O$_2$
0h
3h
6h
14h
rTGM2_v2
TGM2_v2
~63kDa
β-tub
50kDa

Figure 1D

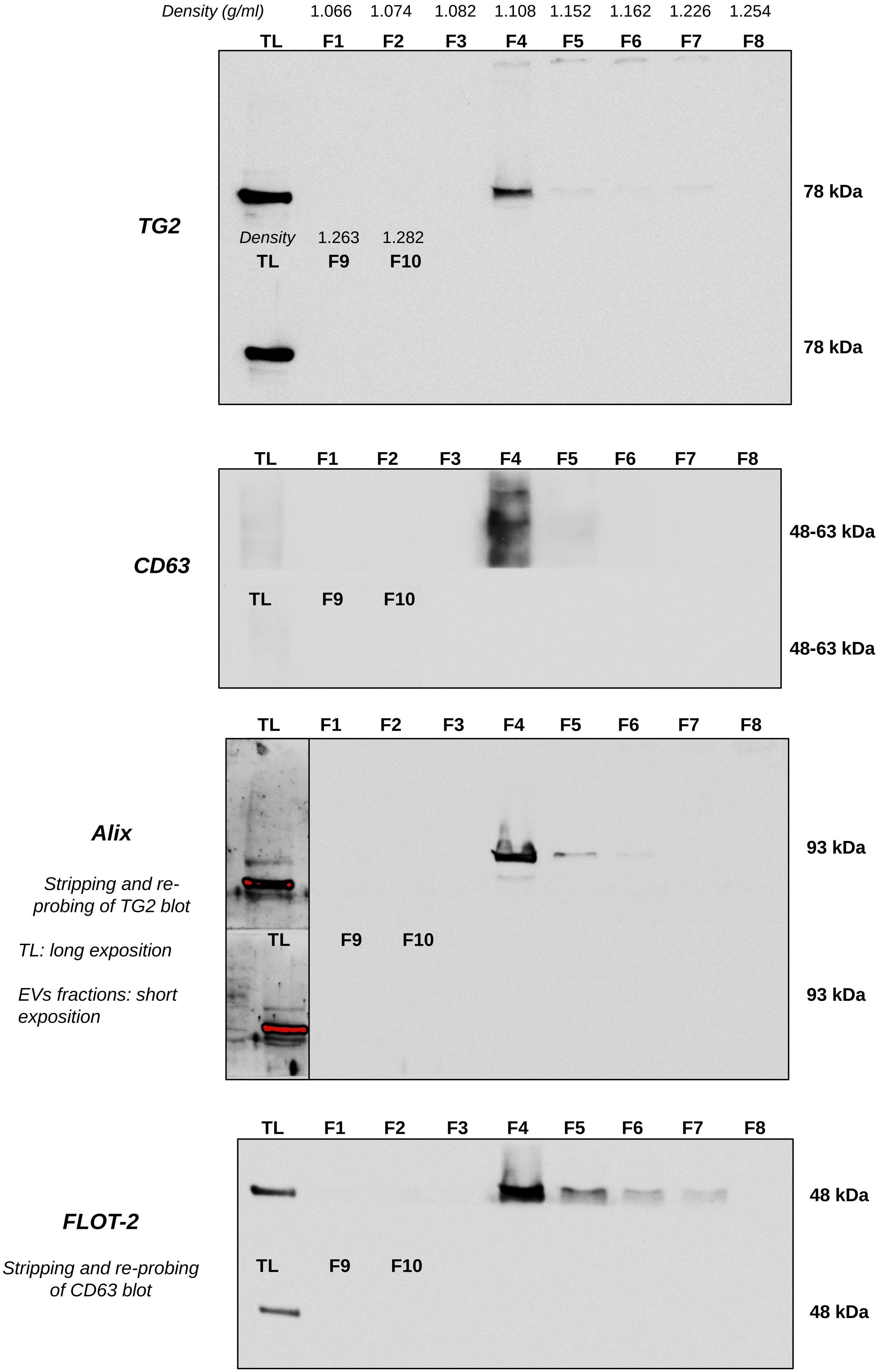

Density (g/ml)
1.066
1.074
1.082
1.108
1.152
1.162
1.226
1.254
TL
F1
F2
F3
F4
F5
F6
F7
F8
TG2
78 kDa
Density
1.263
1.282
TL
F9
F10
78 kDa
TL
F1
F2
F3
F4
F5
F6
F7
F8
CD63
48-63 kDa
TL
F9
F10
48-63 kDa
TL
F1
F2
F3
F4
F5
F6
F7
F8
Alix
Stripping and re-probing of TG2 blot
TL: long exposition
EVs fractions: short exposition
93 kDa
TL
F9
F10
93 kDa
TL
F1
F2
F3
F4
F5
F6
F7
F8
FLOT-2
Stripping and re-probing of CD63 blot
48 kDa
TL
F9
F10
48 kDa

Figure 1G

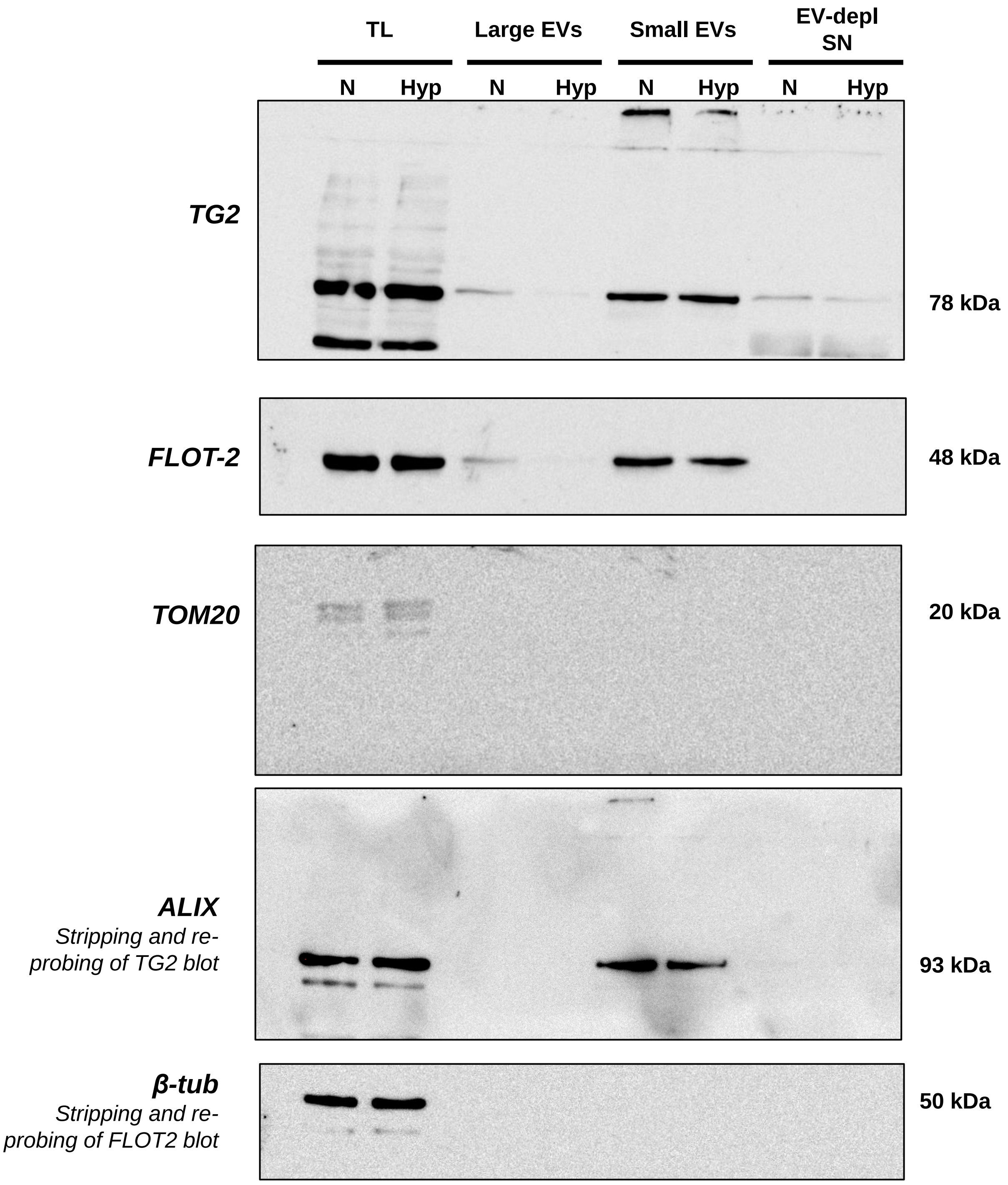
TL
Large EVs
Small EVs
EV-depl SN
N
Hyp
N
Hyp
N
Hyp
N
Hyp
TG2
78 kDa
FLOT-2
48 kDa
TOM20
20 kDa
ALIX
Stripping and re-probing of TG2 blot
93 kDa
β-tub
Stripping and re-probing of FLOT2 blot
50 kDa

Figure 4A

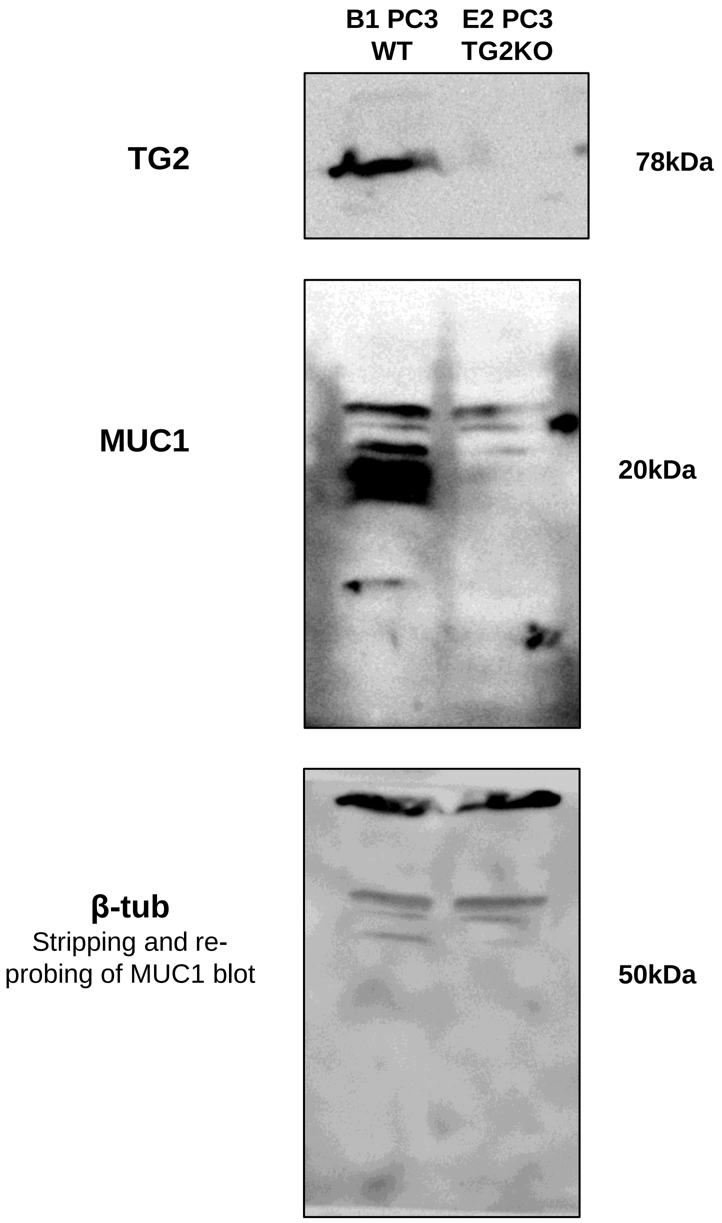
B1 PC3
WT
E2 PC3
TG2KO
TG2
78kDa
MUC1
20kDa
β-tub
Stripping and re-
probing of MUC1 blot
50kDa

Figure 4B

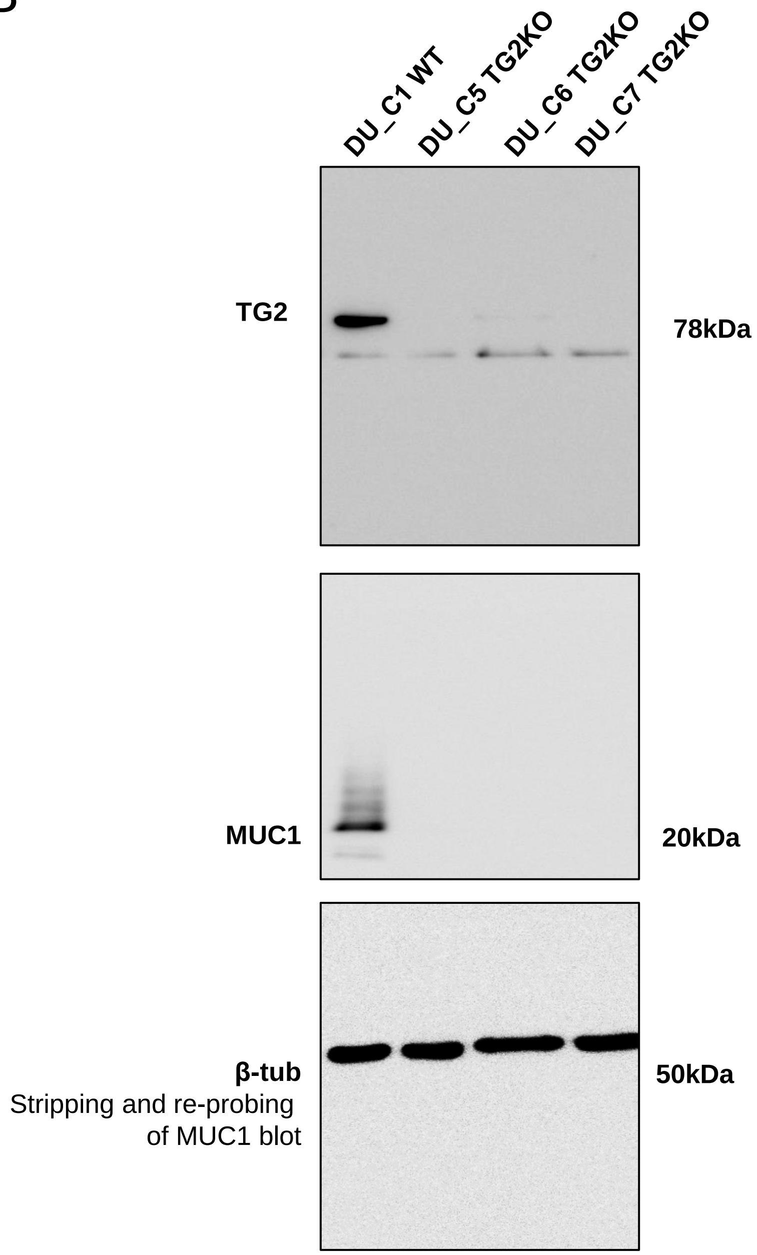

DU_C1 WT
DU_C5 TG2KO
DU_C6 TG2KO
DU_C7 TG2KO
TG2
78kDa
MUC1
20kDa
β-tub
Stripping and re-probing
of MUC1 blot
50kDa

Figure 4G

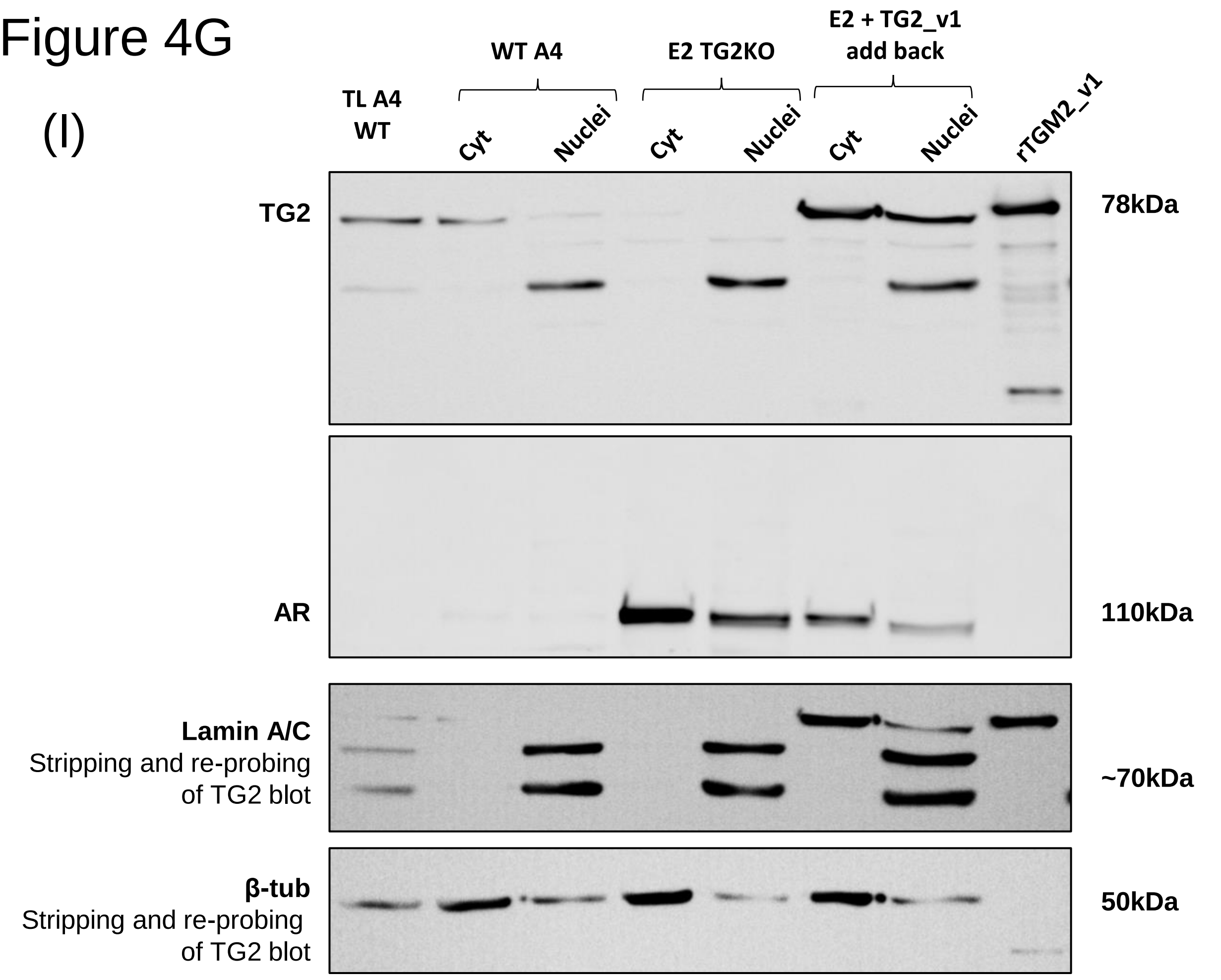
(I)
WT A4
E2 TG2KO
E2 + TG2_v1 add back
TL A4 WT
Cyt
Nuclei
Cyt
Nuclei
Cyt
Nuclei
rTGM2_v1
TG2
78kDa
AR
110kDa
Lamin A/C
Stripping and re-probing of TG2 blot
~70kDa
β-tub
Stripping and re-probing of TG2 blot
50kDa

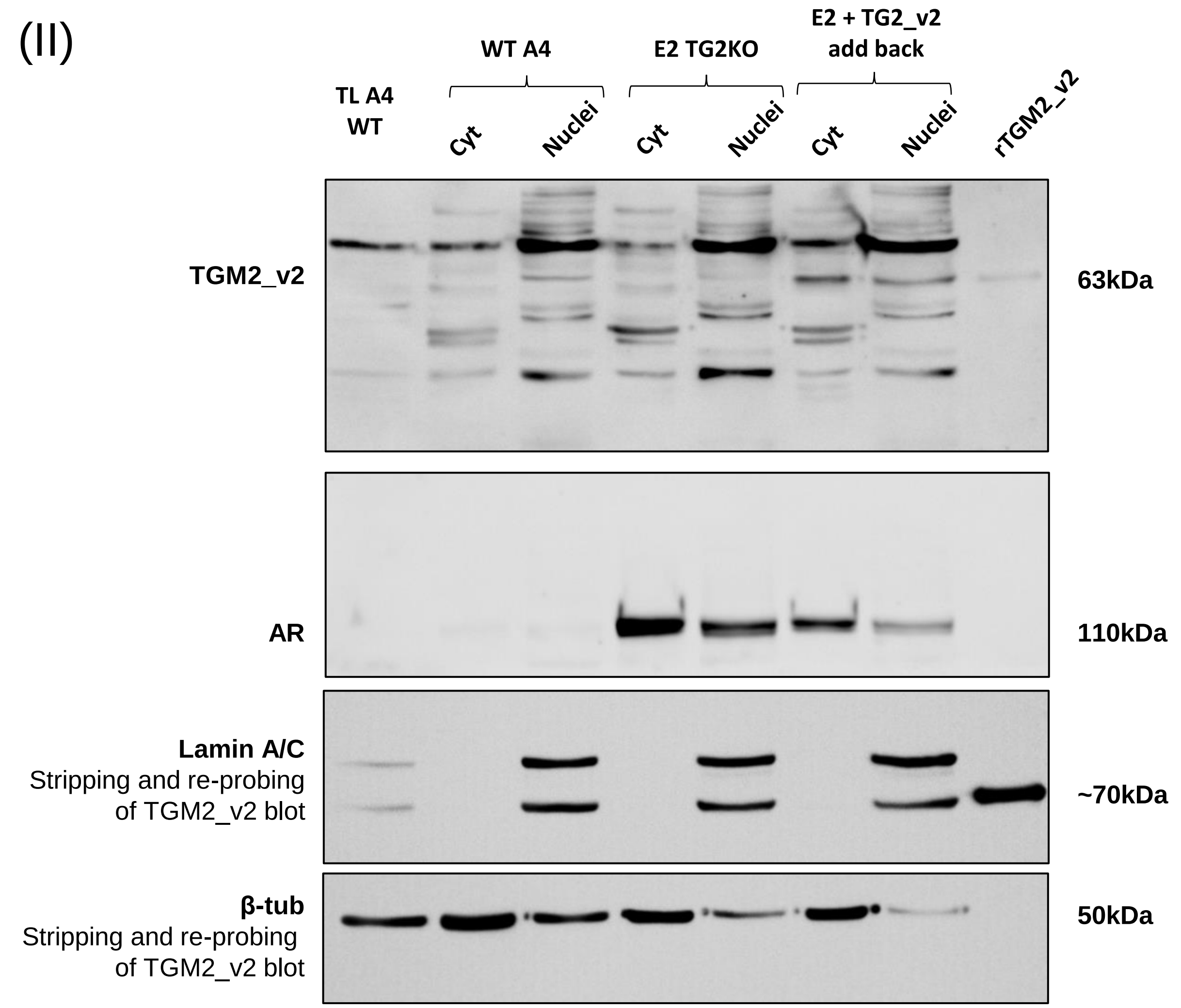
(II)
WT A4
E2 TG2KO
E2 + TG2_v2 add back
TL A4 WT
Cyt
Nuclei
Cyt
Nuclei
Cyt
Nuclei
rTGM2_v2
TGM2_v2
63kDa
AR
110kDa
Lamin A/C
Stripping and re-probing of TGM2_v2 blot
~70kDa
β-tub
Stripping and re-probing of TGM2_v2 blot
50kDa

Supplementary Figure 1

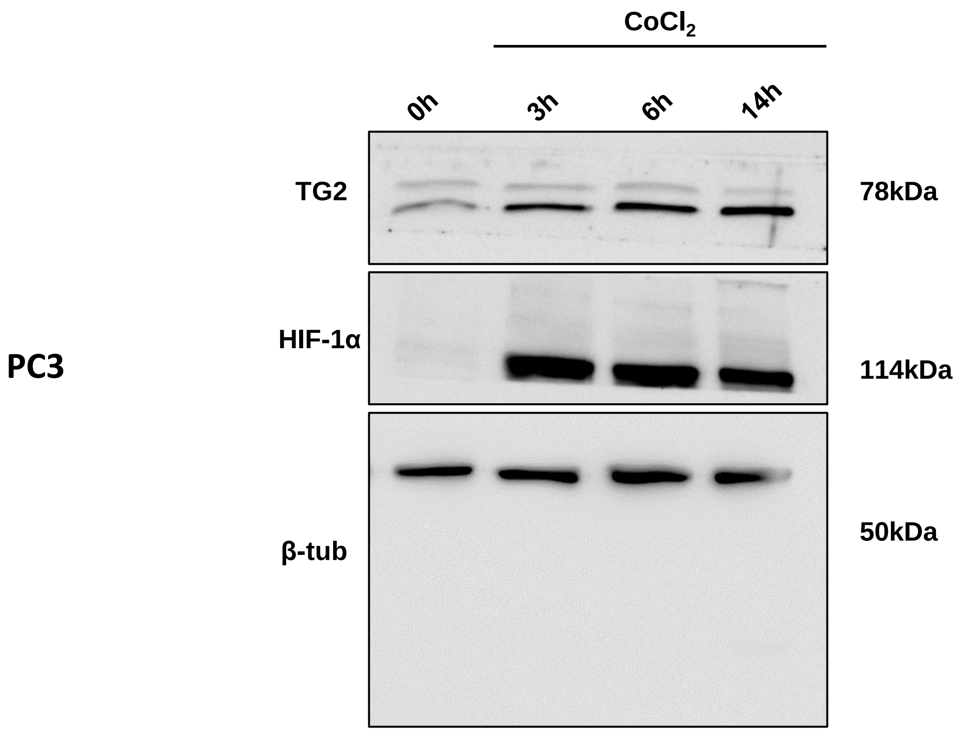

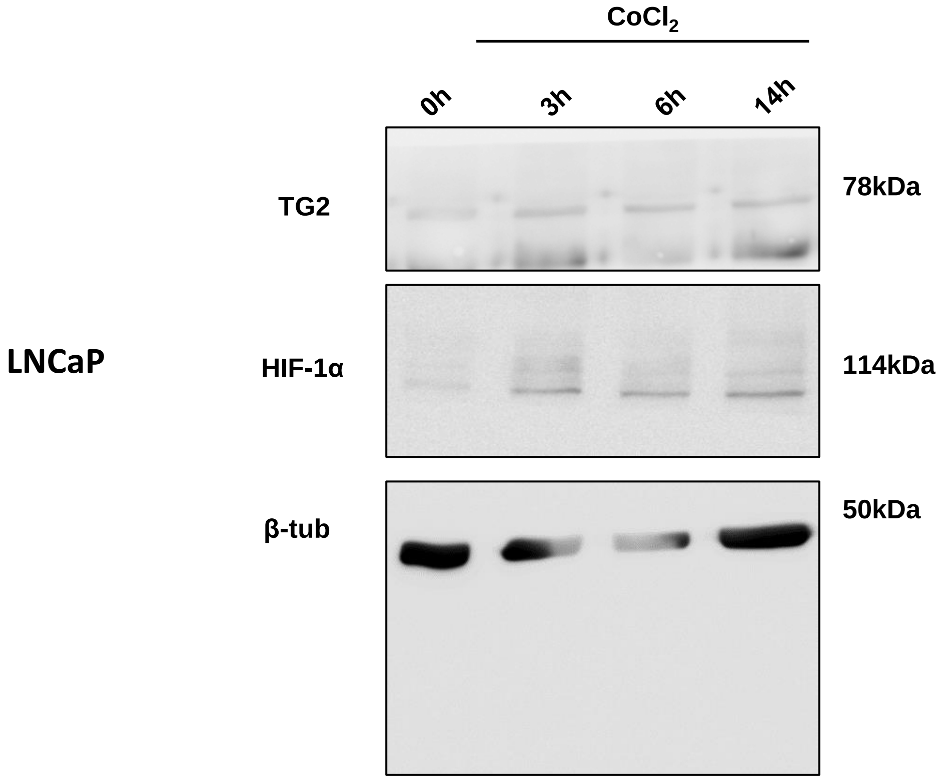

Supplementary Figure 2

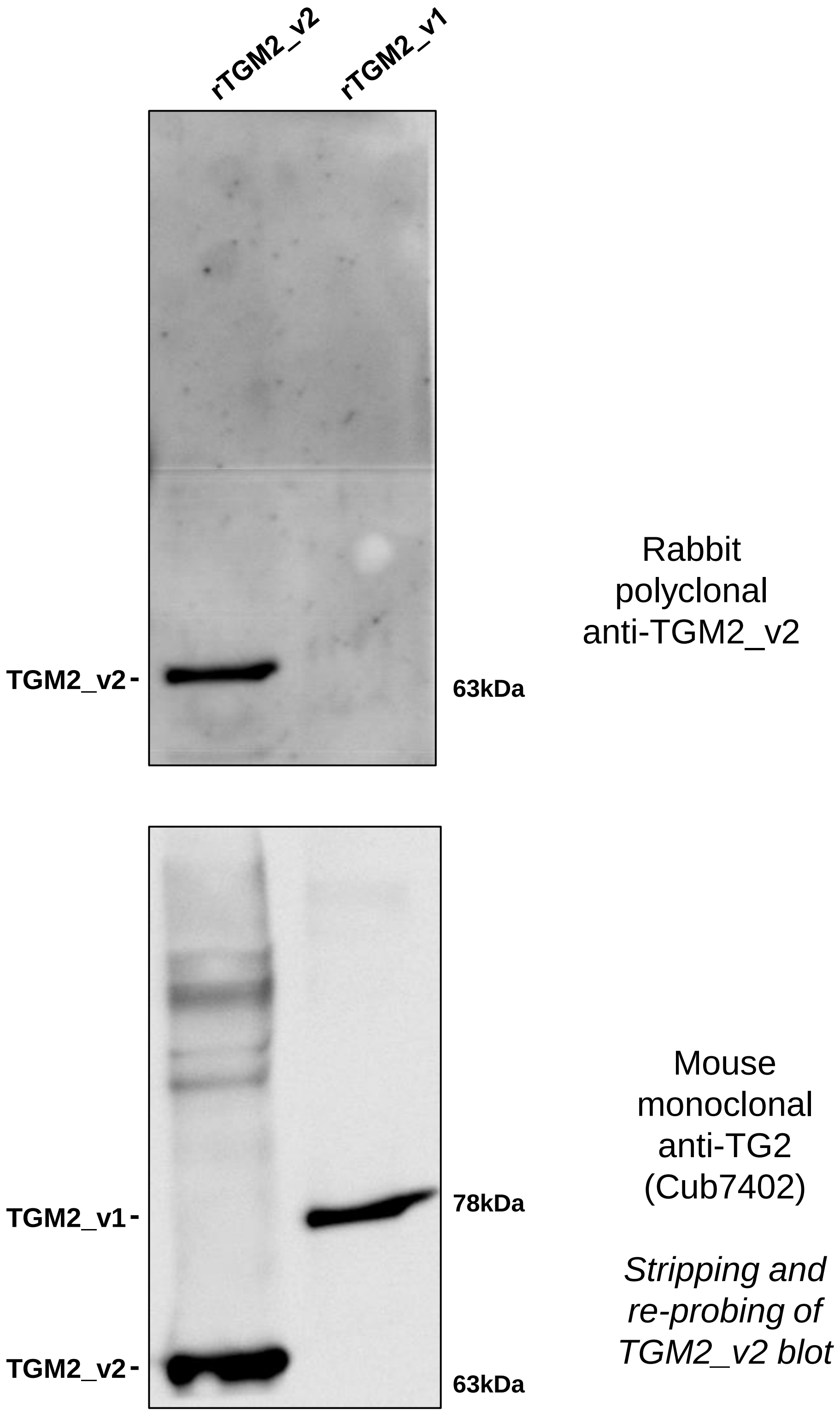

Supplementary Figure 3

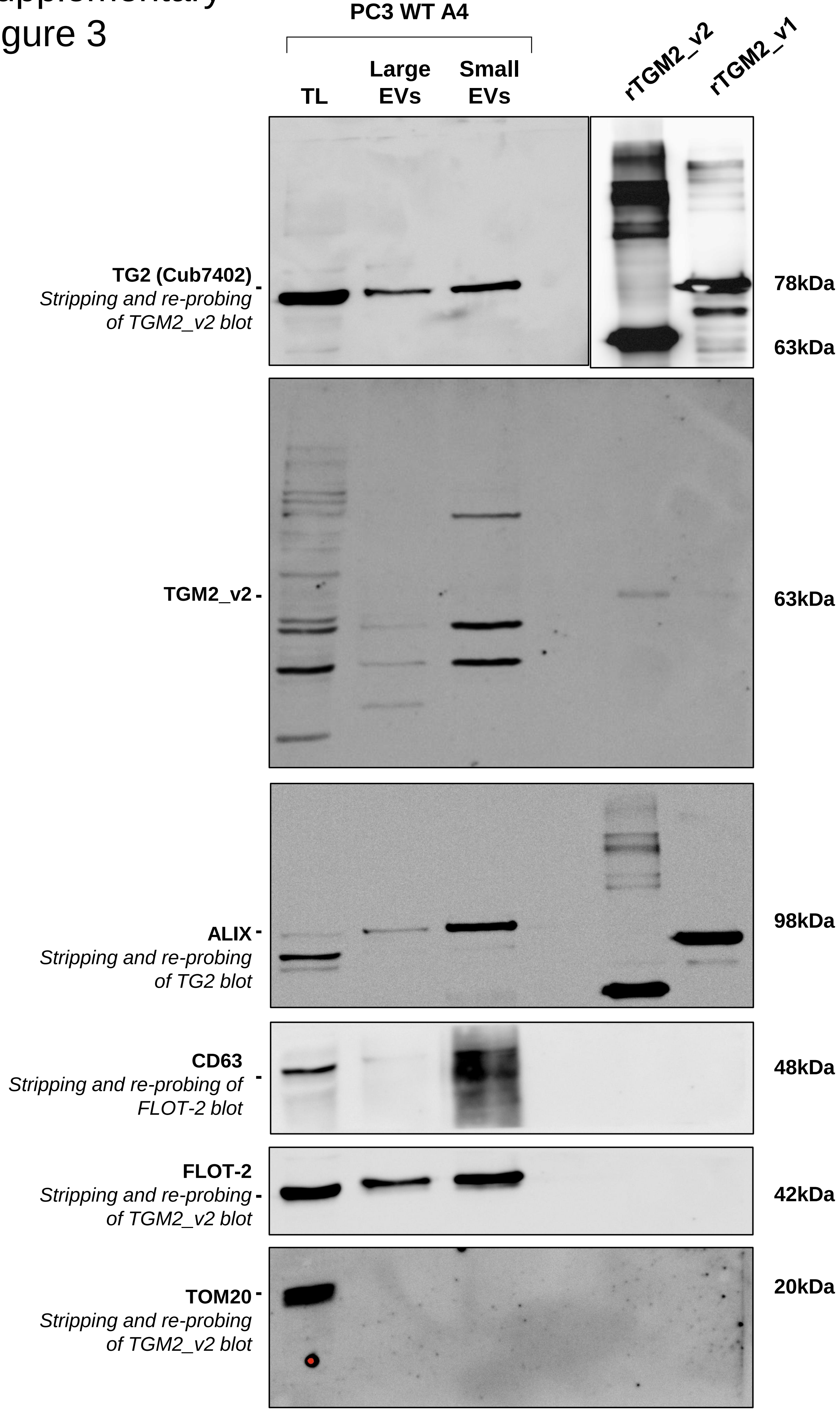

Supplementary Figure 5A

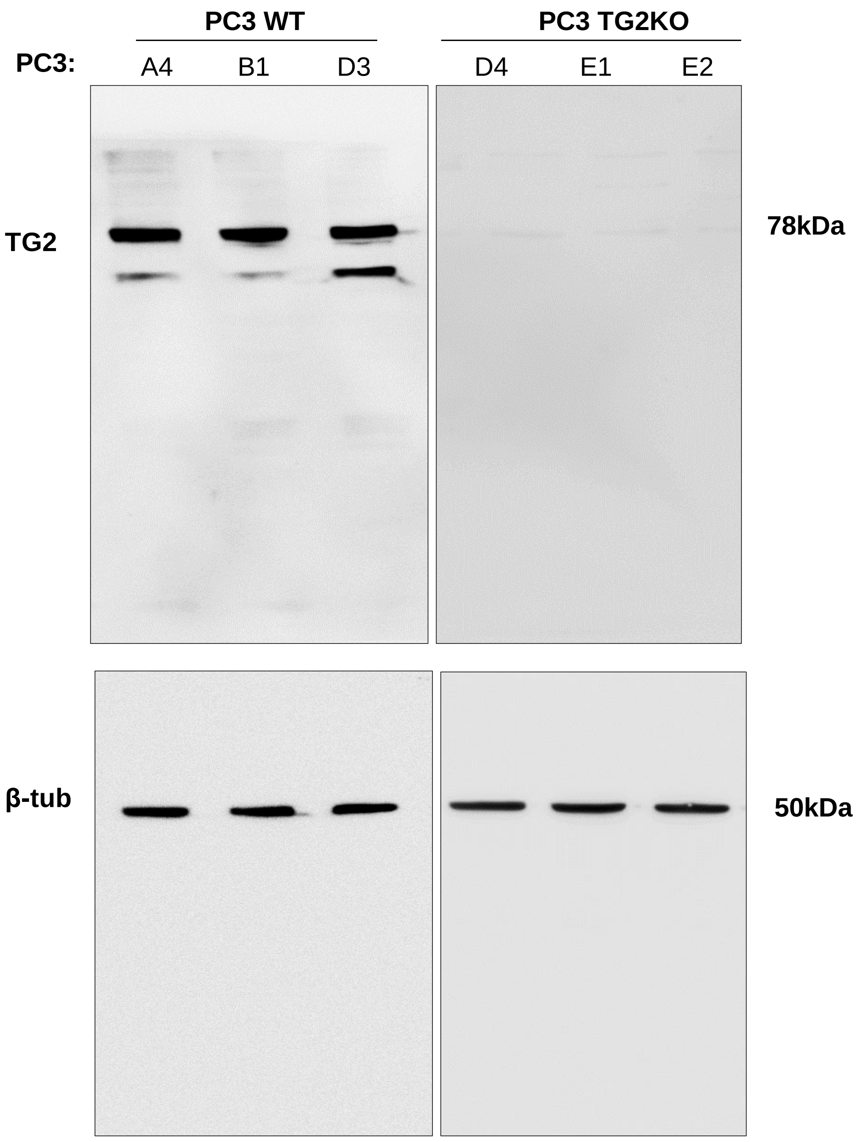

Supplementary Figure 5B

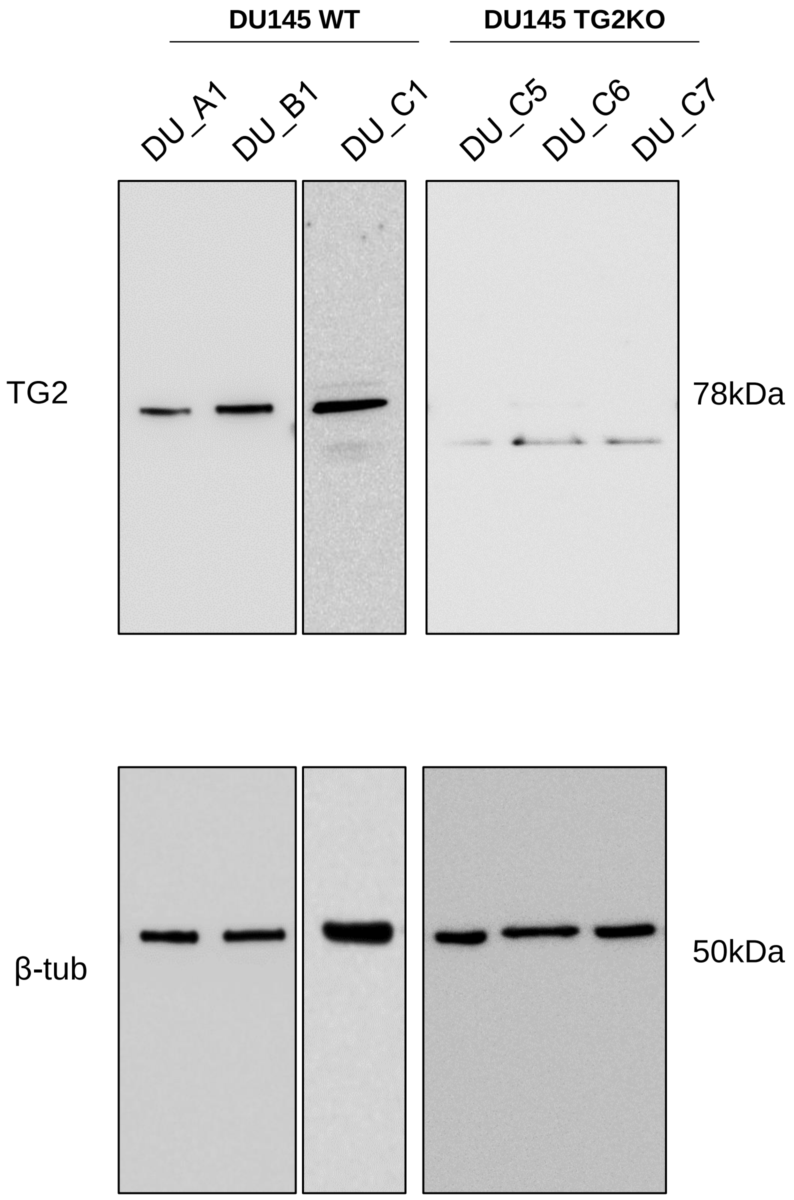

Supplementary Figure 8

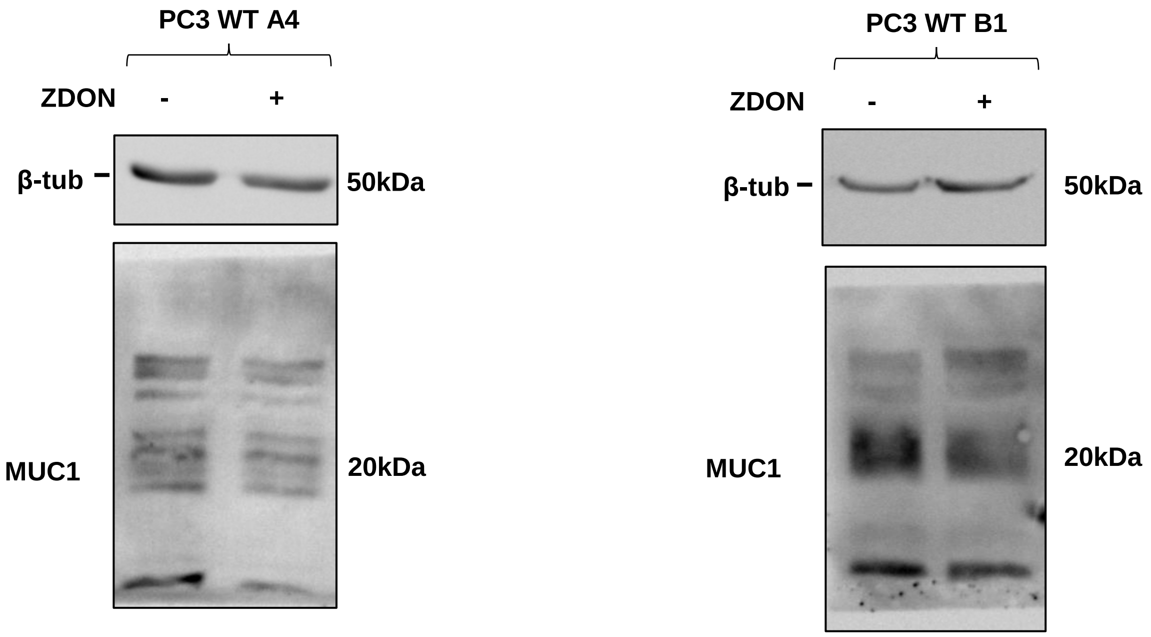

Supplementary Figure 9A

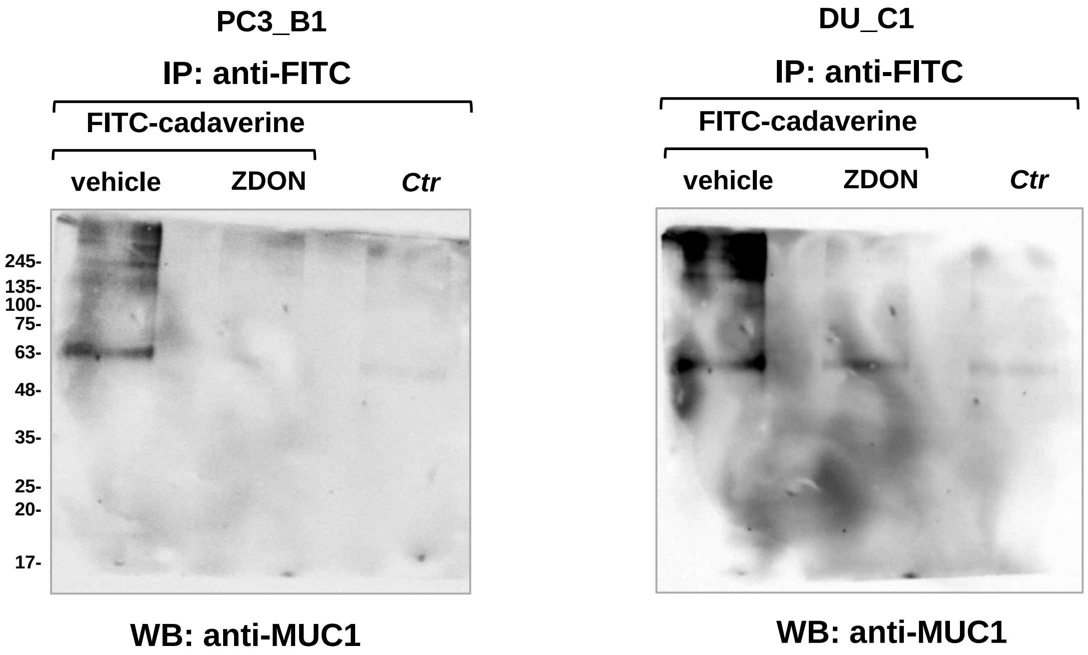

Supplement: Supplementary file 5 — Original Western blots [file 41419_2023_5818_MOESM5_ESM.pdf]
